# Organizational Downsizing, Work Conditions, and Employee Outcomes: Identifying Targets for Workplace Intervention among Survivors

**DOI:** 10.3390/ijerph17030719

**Published:** 2020-01-22

**Authors:** Michael R. Frone, Ann-Renee Blais

**Affiliations:** 1 Department of Psychology, University at Buffalo, The State University of New York, Buffalo, NY 14203, USA; 2 Department of National Defence, Ottawa, ON K1A 0K2, Canada; ann-renee.blais@forces.gc.ca

**Keywords:** organizational downsizing, recession, work conditions, inability to detach, fatigue, negative mood, positive mood, health, work attitudes

## Abstract

This study broadly assesses the association of organizational downsizing to work conditions and employee outcomes, and the extent to which work conditions mediate the association of downsizing to employee outcomes, thereby serving as targets for workplace intervention to reduce the harmful effects of downsizing on surviving workers. The cross-sectional data came from a national probability sample of 2297 U.S. workers. A parallel multiple-mediator model with multiple outcomes was estimated, adjusting for personal, occupational, geographic, and temporal covariates. Exposure to downsizing was the predictor. A set of 12 work conditions, representing four dimensions of the work environment, served as simultaneous mediators (*Work Role:* work demands, role conflict, role ambiguity, and work autonomy; *Interpersonal Relationships:* supervisor aggression, coworker aggression, friendship formation, and dysfunctional leadership; *Rewards:* distributive justice and promotion opportunities; *Security:* job insecurity and employment insecurity). A set of 16 employee consequences, representing five categories of outcomes, served as simultaneous outcomes (*Inability to Detach from Work:* negative work rumination and inability to unwind after work; *Energetic Resource Depletion:* physical, mental, and emotional work fatigue; *Negative Affect:* depression, anxiety, and anger; *Positive Affect:* happiness, confidence, and vigor; *Health:* physical and mental health; *Work Attitudes:* job satisfaction, organizational commitment, and turnover intentions). The results indicated that downsizing had an adverse association with nine of the 12 work conditions (higher levels of work demands, role conflict, supervisor aggression, dysfunctional leadership, job insecurity, and employment insecurity, and lower levels of friendship formation, distributive justice, and promotion opportunities) and all 16 employee outcomes. Moreover, the associations of downsizing to the employee outcomes were indirect, collectively mediated by the nine work conditions. This study provides the broadest evaluation of the deleterious effects of downsizing on U.S. workers surviving a downsizing, identifies affected work conditions that can serve as targets for workplace interventions, and provides insight into why organizational downsizing often fails to deliver anticipated financial and performance benefits to organizations. In terms of serving as targets for workplace intervention, some work conditions meditated the associations of downsizing to a broad set of employee outcomes, whereas other work conditions were specific to certain outcomes. The broad mediators should be targets of any intervention aimed at reducing the adverse effects of downsizing, with additional workplace targets depending on the class of outcomes to be addressed by the intervention.

## 1. Introduction

Organizational downsizing represents the strategic reduction of an organization’s workforce to reduce labor costs, increase profitability, and in times of severe economic shock (e.g., recession), to prevent organizational collapse [1]. Once viewed as an aberration in organizational strategy [2,3], over the prior four decades, downsizing became a widely used reactive strategy to deal with macroeconomic shock and an accepted proactive tool for short-term profit maximization among healthy companies [3,4,5]. Regardless of the reasons for downsizing, it creates two groups of workers: (a) displaced workers who involuntarily lose their jobs (victims) and (b) workers who survived the downsizing (survivors). A large body of research shows that involuntary job loss from downsizing is stressful and results in a variety of adverse outcomes among victims, such as poor physical and mental health and excessive alcohol use [6,7,8,9], as well as scarring effects after reemployment, such as wage penalties and sustained poor mental health [10,11].

Although research also implicates downsizing in detrimental effects on surviving employees, Maertz, Wiley, LeRouge, and Campion [12] pointed out that “there have been few valid direct tests of the supposed negative main effects [of downsizing] on survivor reactions and retention. Instead, most prominent research on survivor reactions has simply examined empirical relationships *among survivors*” (italics in original, p. 277). Therefore, we use a large national sample of U.S. workers that contains downsizing survivors and unexposed workers to address two prominent gaps in this literature. The first gap is the relative lack of research that broadly assesses the work characteristics and employee outcomes that may be affected by organizational downsizing. This lack of broad research leaves primary stakeholders (i.e., employers, labor unions, healthcare providers, and governments) ill-informed regarding the potential impact of downsizing on surviving employees. Whether downsizing has narrow or broad consequences for surviving employees is an issue central to determining policy and the response of primary stakeholders. The second gap is the lack of information on the mediating role of work conditions in the association of organizational downsizing to employee outcomes. The development of policy and the response of stakeholders also is contingent on knowing the work conditions that carry the effect of downsizing on employee outcomes. These work conditions provide targets for workplace intervention that may help mitigate the adverse impact of downsizing on surviving employees.

## 2. Conceptual Model

**Figure 1:**
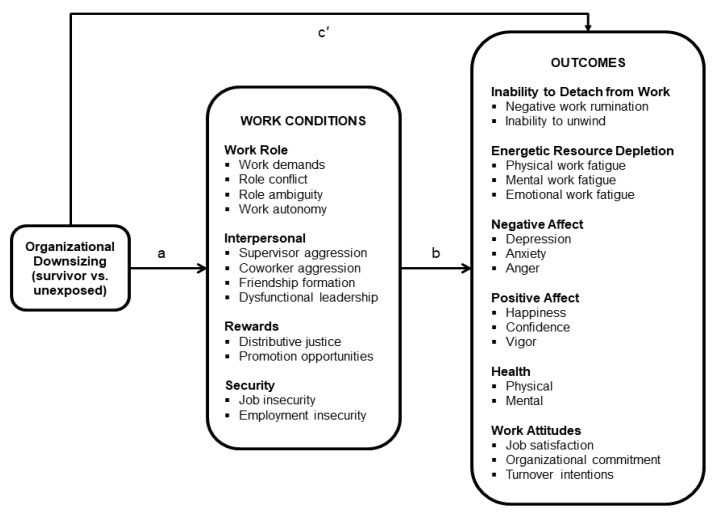
Conceptual model of organizational downsizing, work conditions, and employee outcomes. a = the direct effect of organizational downsizing on work conditions, b = the direct effect of work conditions on the outcomes, and c′ = the direct effect of organizational downsizing on the outcomes.

We use Pearlin and Bierman’s [13] general model of stress to address these gaps and to expand our knowledge of the effects of organizational downsizing on survivors. Pearlin and Bierman’s [13] model proposes that primary stressors can lead to a proliferation of secondary stressors, which subsequently lead to various deleterious outcomes. Building from this general model, the conceptual model of organizational downsizing in Figure 1 leads to three general predictions. First, organizational downsizing represents a macro-organizational primary stressor that can lead to a multitude of secondary workplace stressors characterized by an increase in negative work conditions (e.g., work demands, interpersonal aggression, job insecurity) and a decrease in positive work conditions (e.g., work autonomy, friendship formation, promotion opportunity). Second, downsizing is potentially associated with a broad set of adverse employee outcomes (e.g., inability to detach from work, poor health, negative work attitudes). Third, work conditions will mediate (i.e., explain) the association of downsizing to the adverse employee outcomes. Relatedly, depending on the extent to which the assessed work conditions fully capture the effect of downsizing on employee outcomes, there may or may not be a residual direct association of downsizing with the outcomes.

## 3. Prior Research

### 3.1. Downsizing, Work Conditions, and Employee Outcomes

Although downsizing may affect a broad set of work conditions, few studies have explored this issue, and among those that did, relatively few work conditions were assessed. A study of Finnish municipal employees during a severe recession in the early 1990s found that the extent of downsizing in the surveyed companies (minor, <8%; intermediate, 8–18%; major, >18%) was significantly and positively associated with physical demands and job insecurity; significantly and negatively associated with skill discretion and participation in decision making; and was not significantly related to psychological demands, work autonomy, supervisor social support, and coworker social support [14]. A national study of Norwegian workers in 2003 found that compared with workers unexposed to downsizing, downsizing survivors reported significantly higher levels of work demands and job insecurity, and no difference for work autonomy [15].

A study of Polish workers in 2011 found that compared with workers unexposed to downsizing, downsizing survivors reported significantly higher levels of quantitative work demands and job insecurity; and significantly lower levels of job control (i.e., work autonomy), task clarity (i.e., low role ambiguity), and work-related social support [16]. A study of Korean banking employees explored the impact of downsizing across two banks that differed in the extent of workforce reduction (15% vs. 40%) during a 1997 recession [17]. The results indicated that, among surviving employees, more severe downsizing was associated with lower levels of supervisor support, promotion opportunities, participation in decision making, three types of organizational justice (distributive, procedural, and interpersonal), and job complexity. Finally, a national study of U.S. workers in 1997 found that compared with workers unexposed to downsizing, downsizing survivors reported significantly higher levels of job pressure and coworker social support; and significantly lower levels of work autonomy and organizational support [18].

Employee outcomes have received more attention than work conditions, but most studies have assessed only one or two outcomes. Several studies have shown that organizational downsizing is associated with various employee outcomes, such as higher levels of sickness absence [14,19] and overall work exhaustion [20], as well as lower levels of job satisfaction [20,21], organizational commitment [17,18,22], and physical or mental health [23]. Also, two studies explored the association of downsizing to a broader set of employee outcomes. A study of Polish workers found that compared with unexposed workers, downsizing survivors reported higher levels of stress and turnover intentions; lower levels of job satisfaction, work engagement, and job performance; and no significant difference in overall work exhaustion and sickness absence [16]. A large national study of U.S workers found that organizational downsizing was significantly and negatively related to organizational commitment and perceived performance, significantly and positively related to turnover intentions, and not significantly related to job satisfaction [12].

As shown in Figure 1, we build upon and expand prior research by exploring the association of downsizing to a broader set of work conditions (four general categories—work role conditions, interpersonal relationships, rewards, and security—comprised of 12 constructs) and employee outcomes (six general categories—inability to detach from work, energetic resource depletion, health, negative moods, positive moods, and work attitudes—comprised of 16 constructs). Some of the work conditions and outcomes assessed in the present study overlap with research described earlier, thereby providing replication, and many new work conditions and employee outcomes are assessed in order to extend our understand of the potential impact of organizational downsizing on survivors.

### 3.2. Mediating Role of Work Conditions

Although understanding the work conditions that mediate the association of organizational downsizing to employee outcomes is important because they may serve as potential targets for workplace intervention, surprisingly little research has explored this issue [17]. We identified four studies, described earlier, that tested indirect effects that might inform the development of workplace interventions. A study of Finnish employees [14] reported that downsizing was associated with four (physical demands, skill discretion, participation in decision making, and job insecurity) of the eight work conditions described earlier. Controlling for these four work conditions simultaneously reduced the size of the association between downsizing and sickness absence by 49%, though the direct association of downsizing remained statistically significant, suggesting that other unassessed work conditions may act as mediators [24]. However, the study did not report estimates of the four simultaneous individual indirect effects. Instead, the authors provided evidence suggesting each of the four work conditions acted as a mediator of the association between severity of downsizing and sickness absence if the size of the downsizing association became smaller after statistically controlling for each work condition individually. Because the four work conditions likely were intercorrelated, it is not clear if all four work conditions represent significant mediating variables.

A study of Korean banking employees [17] found that when the seven work conditions described earlier were considered as simultaneous mediating variables, the association of downsizing severity to reduced organizational commitment was fully mediated by three of the seven work conditions: lower levels of promotion opportunities, procedural justice, and job complexity. Knudsen et al.’s [18] national study of U.S. workers found that each of the four assessed work conditions (work demands, work autonomy, coworker support, and organizational support) simultaneously mediated the association between downsizing survivorship and reduced organizational commitment. However, the direct association of downsizing survivorship to reduced organizational commitment remained statistically significant, suggesting that other unassessed work conditions may act as mediators. Finally, a national study of Irish workers reported that work demands mediated the association organizational downsizing to lower levels of job satisfaction and higher levels of overall work exhaustion [20]. However, the direct effects of downsizing to the two outcomes were statistically significant, suggesting that other unassessed work conditions may act as mediators.

Collectively, these four studies exploring the mediating effects of work conditions in the association of organizational downsizing to employee outcomes provide limited information regarding targets for workplace interventions. These studies assessed relatively few work conditions and even fewer employee outcomes. Two studies assessed organizational commitment, one study assessed sickness absence, and one study assessed both job satisfaction and overall work exhaustion.

## 4. The Present Study

We used the model in Figure 1 as a framework to explore four broad research questions. RQ1: Does organizational downsizing have a narrow or broad association with work conditions representing secondary stressors? RQ2: Does organizational downsizing have a narrow or broad association with employee outcomes? RQ3: Which work conditions mediate the association of organizational downsizing to employee outcomes? RQ4: Do the mediating work conditions have general mediating effects across a range of employee outcomes or more narrow mediating effects associated with specific types of outcomes (e.g., inability to psychologically detach from work, health, or work attitudes)? Knowledge of which work conditions have broad or narrow mediating effects would help focus intervention efforts in order to have a maximal impact on reducing the potential deleterious employee outcomes. We address these research questions by comparing workers who were employed by organizations that had layoffs during the preceding year (survivors) to workers who reported that their employer had no layoffs (unexposed), using data from a large national probability sample of U.S. wage and salary workers.

## 5. Materials and Methods

### 5.1. Sample and Study Design

Data came from 2975 U.S. workers who took part in a national random-digit-dial telephone survey, called the National Survey of Work Stress and Health, conducted from December 2008 to April 2011. The study design is described in more detail elsewhere [25]. To be included in the present analyses, participants had to meet three sequential selection criteria: (a) answered the question about company downsizing (510 workers were excluded because the question was added after the survey was in the field), (b) were wage and salary workers (144 owner/operators were not included), and (c) had data on all covariates described later (24 wage and salary workers were missing information on the covariates). These selection criteria resulted in a final sample of 2297 workers. The study protocol (Study #2448) was approved by the University at Buffalo’s Human Research Institutional Review Board. All study participants provided verbal informed consent before participating in the study.

#### 5.1.1. Sampling Weights

The participants were weighted using sampling weights to better generalize to the target population of U.S. wage and salary workers. The sampling weights adjust for differential probabilities of selection, nonresponse, and noncoverage, and are described in more detail elsewhere [25].

#### 5.1.2. Respondent Characteristics

The respondent characteristics (constructs used as covariates) are described in Table 1. 

### 5.2. Measures

Descriptive statistics (means and standard deviations) and correlations for all variables are presented in Appendix S1.

#### 5.2.1. Organizational Downsizing

Participants reported if their employer had laid off employees during the preceding 12 months (not including usual seasonal layoffs). Responses were scored as 0 (no) and 1 (yes).

#### 5.2.2. Work Conditions

*Work demands* were assessed with six commonly used items [26,27,28]. Example items are as follows: During the past 12 months, how often did you have too much work to do? and During the past 12 months, how often did your job require you to work under time pressure? Response anchors ranged from 0 (never) to 4 (every day). Coefficient alpha was 0.87.

*Role conflict* was assessed with three items—two items from Peterson et al. [29] and one item from House, Schuler, and Levanoni [30]. *Role ambiguity* was assessed with four items developed by House et al. [30]. An example role conflict item *is I often have to meet the conflicting demands from various people at work*. An example role ambiguity item is *My work responsibilities are clearly defined* (reverse scored). Response anchors ranged from 1 (strongly disagree) to 4 (strongly agree). Coefficient alpha was 0.86 for role conflict and 0.82 for role ambiguity.

Work autonomy was assessed with six items adapted from Morgeson and Humphrey [31]. Example items are I can make my own decisions about how to schedule my work, and I can make decisions about what methods I use to complete my work. Response anchors ranged from 1 (strongly disagree) to 4 (strongly agree). Coefficient alpha was 0.86.

Supervisor and coworker aggression were each assessed with three parallel items adapted from prior research [32,33]. Items for supervisor aggression were Thinking back over the past 12 months, how often has a supervisor done any of the following to you? Was rude or talked down to you? Made negative comments about your intelligence, competence, or productivity? Insulted you or called you names in front of other people? Response anchors ranged from 0 (never) to 4 (every day). Coefficient alpha was 0.82 for supervisor aggression and 0.80 for coworker aggression.

*Friendship formation*—the extent to which respondents formed strong friendships at work—was assessed with a three-item measure developed for this study. An example item is *I feel close to some of the people I work with*. Response anchors ranged from 1 (strongly disagree) to 4 (strongly agree). Coefficient alpha was 0.90. 

*Dysfunctional leadership* was assessed with items assessing passive and undermining leadership. Of the five items assessing passive leadership—two items were adapted from Den Hartog, Van Muijen, and Koopman [34], two were from Pearce and Sims [35], and one item was developed for this study. An example item is *Your supervisor tends to be unavailable when staff need help with a problem*. Undermining leadership was assessed with five items [36]. An example item is *Your supervisor changes goals without telling you*. Response anchors for all 10 items ranged from 1 (strongly disagree) to 4 (strongly agree). Because the passive and undermining leadership measures correlated highly (r = 0.75), the items were combined into an overall measure of dysfunctional leadership. Coefficient alpha for the overall measure was 0.92.

*Distributive justice*—the extent to which individuals are rewarded fairly, given their efforts and contributions—was assessed with four items [37]. An example item is *My rewards reflect the effort I put into my work*. Response anchors ranged from 1 (strongly disagree) to 4 (strongly agree). Coefficient alpha was 0.94.

*Promotion opportunity* was assessed with two items adapted from Spector [38], and one item developed for this study. An example item is *There is little chance of promotion on my job* (reverse scored). Response anchors ranged from 1 (strongly disagree) to 4 (strongly agree). Coefficient alpha was 0.85.

*Job and employment insecurity* were each assessed with three items [39]. Job insecurity represents the perceived likelihood of involuntarily losing one’s current job, whereas employment insecurity represents the perceived likelihood of not finding comparable new employment in the event of job loss [40]. An example job insecurity item is *I am not really sure how long my present job will last.* An example employment insecurity item is *If I lost my present job, I would probably be unemployed for a long time*. The response anchors ranged from 1 (strongly disagree) to 4 (strongly agree). Coefficient alpha was 0.80 for job insecurity and 0.85 for employment insecurity.

#### 5.2.3. Employee Outcomes

*Negative work rumination*, which represents preoccupation with and repetitive thoughts focused on negative work experiences that may extend beyond the workday, was assessed with five items [25]. An example item is *How often do you replay negative work events in your mind even after you leave work?* The response anchors ranged from 0 (never) to 3 (often). Coefficient alpha was 0.91.

*Inability to unwind* was assessed with the following single item developed for this study: *During the past 12 months, how often did you find it difficult to unwind and relax after you leave work?* The response anchors ranged from 0 (never) to 4 (every day).

*Physical, mental, and emotional work fatigue* were each assessed with six parallel items from the Three-Dimensional Work Fatigue Index (3D-WFI) [41]. Example emotional fatigue items are *During the past 12 months, how often did you feel emotionally exhausted at the end of the workday?*; and *During the past 12 months, how often did you want to emotionally shut down at the end of the workday?* Response anchors ranged from 0 (never) to 4 (every day). Coefficient alpha was 0.94 for physical work fatigue, 0.95 for mental work fatigue, and 0.95 for emotional work fatigue.

Three dimensions of negative and positive affect were assessed by asking how often the participants experienced each emotion during the prior 12 months. Each negative and positive emotion was assessed with three adjectives: *depression* (depressed, sad, gloomy), *anxiety* (nervous, anxious, worried), *anger* (hostile, furious, angry), *happiness* (joyful, happy, cheerful), *confidence* (confident, proud, strong), and *vigor* (lively, active, energetic). The emotion adjectives were taken from the Brunel mood scale [42] and the PANAS-X [43]. Response anchors for the emotion adjectives ranged from 0 (never) to 3 (often). Coefficient alpha was 0.75 for depression, 0.74 for anxiety, 0.69 for anger, 0.81 for happiness, 0.68 for confidence, and 0.77 for vigor.

Physical and mental health were each assessed with two-items [44]. Physical health was assessed with the following two items: In general, would you say your physical health is poor, fair, good, very good, or excellent?; and In general, compared to most (men/women) your age, is your physical health much better, somewhat better, about the same, somewhat worse, or much worse? (reverse-scored). Mental health was assessed with parallel items substituting mental or emotional health for physical health. The item responses were scored from 1 (poor/much worse) to 5 (excellent/much better). Coefficient alpha was 0.74 for physical health and 0.78 for mental health.

*Job satisfaction* was assessed with three items from the Michigan Organizational Assessment Questionnaire [45]. An example item is *All in all, I am satisfied with my job*. Response anchors ranged from 1 (strongly disagree) to 4 (strongly agree). Coefficient alpha was 0.91.

*Affective organizational commitment* was assessed with three items from Meyer and Allen’s revised measure [40,46]. An example item is *This organization has a great deal of personal meaning to me*. Response anchors ranged from 1 (strongly disagree) to 4 (strongly agree). Coefficient alpha was 0.88.

*Turnover intentions* were assessed with three items [47]. An example item is *I am seriously thinking about quitting my job*. Response anchors ranged from 1 (strongly disagree) to 4 (strongly agree). Coefficient alpha was 0.85.

#### 5.2.4. Covariates

Several covariates were included in the analyses to adjust for potential compositional differences in the samples of downsizing survivors and unexposed employees (see Table 1 for more detail): *gender* (0 = women, 1 = men), *race* (0 = White, 1 = minority), *age* (in years), *level of formal education* (10 ordinal categories, scored 1 to 10), *total annual family income from all sources* (in units of 10,000 U.S. dollars), *U.S. Census geographic divisions* (8 dummy variables, scored 0 and 1, from 9 nominal categories with New England as the referent category), *occupation* (8 dummy variables, scored 0 and 1, from 9 aggregated nominal categories with management/business/financial occupations as the referent category, based on the Standard Occupational Classification system), *industry* (11 dummy variables, scored 0 and 1, from 12 aggregated nominal categories with agriculture/forestry/mining/construction industries serving as the referent category, based on the North American Industry Classification System), *employer has more than one work location* (0 = no, 1 = yes), *number of employees at the respondent’s work location* (11 ordinal categories, scored 1 to 11), *number of total employees in organization* (11 ordinal categories, scored 1 to 11), *job tenure* (in years), *number of weekly work hours*, *seasonal job* (0 = no, 1 = yes), *precarious employment* (0 = no, 1 = yes), *union membership* (0 = no, 1 = yes), and the *calendar quarter in which the respondent was interviewed* (nine ordinal quarters from 1st quarter 2009 to 1st quarter 2011 were scored 1 to 9).

### 5.3. Data Analysis

Before discussing the estimation and results of the structural model shown in Figure 1, we report a confirmatory factor analysis (CFA), estimated using M*plus* [48], to verify the factor structure of the 27 multi-item constructs shown in Figure 1. The downsizing and inability to unwind variables were excluded because they were assessed with single items. The indicator items were treated as ordinal, and the analysis used a robust weighted least squares estimator (WLSMV). The analysis also used sampling weights and took into account missing data in the items. 

To assess overall CFA model fit, we report the model chi-square with its degrees of freedom, and because this statistic can be overly sensitive to large samples [49,50], we also report the following approximate fit indices: (a) the comparative fit index (CFI), (b) the Tucker-Lewis index (TLI), and (c) the root mean square error of approximation (RMSEA) and its 90% confidence interval. Excellent model fit is suggested when CFI ≥ 0.95, TLI ≥ 0.95, and RMSEA ≤ 0.06 [51]. 

The correlated 27-factor model with 104 indicator variables provided an excellent fit to the data: χ^2^ (4901; *n* = 2297) = 6493.60, *p* < 0.001; CFI = 0.988; TLI = 0.987; and RMSEA = 0.012 (90% CI [0.011, 0.013]). Also, the standardized factor loadings shown in Appendix S2 revealed that each indicator variable loaded highly on its intended latent construct.

The structural model shown in Figure 1 was estimated using M*plus* [48] to explore RQs 1–4. All work conditions and employee outcomes were modeled simultaneously, which represents a parallel multiple-mediator model [52,53] with multiple outcomes. The multiple work conditions that served as parallel mediators were allowed to correlate among themselves, as were the multiple outcomes [53]. The covariates shown in Table 1 were included in the analysis as exogenous variables that correlated among themselves and with the downsizing variable and were predictors of each work condition and employee outcome. Maximum likelihood estimation and sampling weights [48] were used, coupled with full information maximum likelihood procedures to address missing data in the work conditions and employee outcomes [48,54]. Finally, robust nonparametric bootstrap standard errors are reported for all model coefficients and the significance of individual indirect effects was based on 95% bias-corrected bootstrap confidence intervals. All bootstrap standard errors and confidence intervals were based on 5000 bootstrapped samples [52].

## 6. Results

### 6.1. Research Question 1: Association of Downsizing with Work Conditions

Table 2 displays the effect of downsizing on each of the 12 work conditions. Downsizing had significant positive associations with work demands, role conflict, supervisor aggression, dysfunctional leadership, and job and employment insecurity. Conversely, it had significant negative associations with friendship formation, distributive justice, and promotion opportunities. Overall, downsizing was significantly related to nine of the 12 work conditions, providing evidence for its broad impact on the work environment.

### 6.2. Research Question 2: Association of Downsizing with Employee Outcomes

Table 3 shows the total effect of downsizing on the 16 employee outcomes. It also shows the decomposition of each total effect into the total indirect effect and the direct effect of organizational downsizing on each outcome. In standard path analytic nomenclature, a total effect (c) is the sum of the total indirect effect (a × b; i.e., the sum of all individual indirect effects across the 12 work conditions) and the direct effect (c′) (see Figure 1) [52,55,56]. Although the total effects are reported, we focus on the total indirect effects to determine if organizational downsizing is associated with each employee outcome. We do this for two reasons. First, if the direction of the direct effect (c′) and the total indirect effect (a × b) are of opposite sign, the size of the total effect can be suppressed [24,56]. Second, in the case of full mediation (no significant direct effect, c′), where the total effect (c) is approximately equivalent to the total indirect effect (a × b), the statistical power of the total indirect effect is higher than that of the total effect [55,56]. Both of these situations can result in a total effect that is not statistically significant, though the total indirect effect is statistically significant.

The results in Table 3 indicate that downsizing had significant total indirect effects on all 16 employee outcomes. More specifically, downsizing was positively related to all variables representing the inability to detach from work, energy depletion, and negative affect, as well as to the work attitude variable intentions to turnover. Conversely, it was negatively associated with all variables representing positive affect and health, as well as with the work attitudes of job satisfaction and organizational commitment. Downsizing exhibited a significant direct effect with only one of the 16 outcomes—the experience of anger. Collectively, these findings provide support for a broad impact of downsizing on employee outcomes and that, except for anger, these associations were mediated fully by the nine significant work conditions discussed earlier.

### 6.3. Research Questions 3 and 4: Work Conditions as Mediators

Table 4 shows the individual indirect effects of downsizing involving each outcome, where the bootstrap confidence interval did not include zero, whereas Table 5 summarizes these indirect effects by work condition. Table 4 shows that although downsizing was indirectly associated with all 16 outcomes (see Table 3), the pattern of mediated effects differed across the work conditions. This is seen more clearly in the summary provided in Table 5, which shows that the mediating effect of some work conditions on employee outcomes is narrow. Reduced promotion opportunities only mediated the associations between downsizing and the three work attitudes, and supervisor aggression only had mediating effects on the three energy depletion outcomes and poor mental health. On the other hand, several other work conditions, including friendship formation, dysfunctional leadership, distributive justice, and job and employment insecurity, had broader mediating effects across a wide range of outcomes, representing all six categories of employee outcomes. Finally, there was one unique pattern of results involving employment insecurity. Downsizing was significantly related to higher levels of employment insecurity and other poor working conditions, which resulted in a wide range of adverse outcomes. However, downsizing was indirectly and negatively, rather than positively, related to turnover intentions via employment insecurity, suggesting that some employees may feel unable to leave the poor work conditions and the resulting adverse outcomes associated with downsizing.

## 7. Discussion

The goals of this study were to determine (a) if organizational downsizing has a narrow or broad impact on work conditions, (b) if organizational downsizing has a narrow or broad impact on employee outcomes, (c) if work conditions mediate the association of organizational downsizing to employee outcomes, and (d) if the mediating work conditions have narrow or general effects across a range of employee outcomes. As discussed in more detail below, the present results extend prior research by showing that organizational downsizing had a broad impact on both work conditions and employee outcomes. Moreover, nine of the 12 work conditions collectively mediated the association of downsizing to all 16 employee outcomes, representing both broad and narrow mediational processes. These work conditions represent potential targets for multicomponent workplace interventions aimed at mitigating the broad set of harmful outcomes.

### 7.1. Association of Downsizing with Work Conditions

Downsizing had an adverse association with nine of the 12 work conditions explored in this study, which represented all four groups of work conditions: work role (work demands, role conflict), interpersonal (supervisor aggression, friendship formation, dysfunctional leadership), rewards (distributive justice, promotion opportunities), and security (job insecurity, employment insecurity). These results support and extend past research. In terms of work role conditions, our results support prior findings that downsizing is positively associated with work demands [14,15,16,18]. The results extend prior research by showing that downsizing is positively associated with role conflict. Only one prior study examined and found a positive association between downsizing and role ambiguity [16], although the present study did not replicate this association. Of the five prior studies that explored work autonomy, there was some support in four studies for a negative association between downsizing and work autonomy [14,16,17,18], whereas one study failed to find an association [15]. Consistent with the study by Østhus [15], the present study also failed to find a significant association between downsizing and work autonomy.

Prior research looking at downsizing and interpersonal work conditions focused exclusively on work-related social support [14,16,17,18]. However, these studies provided inconsistent evidence for an association. Two studies reported a significant *negative* association between downsizing and either overall support [16] or supervisor support [17]. In contrast, one study reported a *positive* association between downsizing and coworker support [18], and one study failed to find an association between downsizing and both supervisor and coworker support [14]. In the present study, we extended prior research by exploring the association of downsizing to a new set of interpersonal work conditions that may more broadly represent the interpersonal climate at work: coworker aggression, supervisor aggression, friendship formation, and dysfunctional leadership. We failed to find a significant association between downsizing and coworker aggression. However, downsizing was significantly and positively associated with two negative supervisor behaviors—exhibiting psychological aggression and dysfunctional leadership (passive supervision and undermining the performance of direct reports). We also documented a significant negative association between downsizing and reports of friendship formation. Compared to unexposed employees, downsizing survivors were less likely to report feeling close to coworkers or that they considered some coworkers as close friends. Downsizing may result in interpersonal distancing because it is possible that more coworkers may lose their jobs, which may have a broad effect on coworker communication and workplace support. Overall, the interpersonal work conditions assessed in this study may be more proximal outcomes of downsizing than social support and better represent interpersonal deterioration during downsizing.

In terms of reward characteristics, only one prior study examined distributive justice (fairness of rewards) and promotion opportunities [17]. Consistent with that study, we found that downsizing was significantly related to lower levels of both distributive justice and promotion opportunities. Finally, in terms of work-related security, downsizing should increase concerns about job insecurity among survivors. Each of the three prior studies exploring this association reported a significant positive association between downsizing and job insecurity [14,15,16]. However, insecurity over the continuity of employment extends beyond fear of job loss (i.e., job insecurity) and includes fear of not being able to obtain another job in the event of job loss (employment insecurity) [40]. Our results both support prior research by showing that downsizing was significantly and positively related to job insecurity and extend this body of research by showing that downsizing also was significantly and positively related to employment insecurity. Although employment insecurity has received little research attention compared with job insecurity [40], as noted later, it also acted as a mediator of the associations of downsizing to employee outcomes along with job insecurity.

### 7.2. Association of Downsizing with Employee Outcomes

As discussed earlier, prior research has shown that downsizing is associated with several deleterious outcomes. However, most studies assessed no more than two potential outcomes, and the range of outcomes across studies was limited. The present results revealed that downsizing had a broad adverse association with 16 employee outcomes. Our study supports prior findings that organization downsizing is positively associated with overall work exhaustion [20] and turnover intentions [12,16] and is negatively associated with job satisfaction [16,20,21], organizational commitment [12,17,18,22], and physical and mental health [23]. More importantly, the present study provides new evidence that organizational downsizing is positively related to two dimensions of employees’ inability to detach from work (negative work rumination and inability to relax), with three specific forms of energy depletion at work (physical, mental, and emotional work fatigue), and three dimensions of negative affect (depression, anxiety, and anger), and that downsizing is negatively related to three dimensions of positive affect (happiness, confidence, and vigor).

In addition to demonstrating the broad impact of organizational downsizing on surviving employees, the present results showed that downsizing was indirectly associated with each of the 16 outcomes via some combination of the nine work conditions. Finally, the only direct association observed was between downsizing and anger, suggesting that unassessed work conditions might mediate this association or that anger represents a direct, visceral response to the experience of organizational downsizing that is not mediated fully by work conditions.

### 7.3. Work Conditions as Mediators

Only four prior studies examined work conditions as mediators of the association of organizational downsizing to employee outcomes [14,17,18,20]. As noted earlier, these studies provided limited evidence for mediation by work outcomes to three employee outcomes. More importantly, three of the four studies [14,18,20] found a direct association of downsizing to the outcomes examined, suggesting that unassessed work conditions may act as mediators. The present study found that some combinations of the nine out of 12 work conditions mediated the association of downsizing to the 16 employee outcomes. As discussed earlier, we found only one significant direct association of downsizing to employee anger.

There were several noteworthy patterns of mediational effects. Five of the nine work conditions (friendship formation, dysfunctional leadership, distributive justice, job insecurity, and employment insecurity) each mediated the association of downsizing to a broad set of employee outcomes, representing from four to five of the six outcome categories. In contrast, four work conditions had more specific mediating effects. Work demands only had mediating effects for outcomes representing an inability to detach from work (negative work rumination and inability to unwind), energy depletion (physical, mental, and emotional work fatigue), and negative affect (depression, anxiety, and anger). Supervisor aggression acted as a mediator for energy depletion (physical, mental, and emotional work fatigue) and mental health. The mediating effect of promotion opportunities was constrained to the three work attitudes (job satisfaction, organizational commitment, and turnover intentions). Finally, the least import work condition was role conflict, which meditated only three outcomes showing no coherent theme (inability to unwind, emotional work fatigue, turnover intentions).

Finally, there was one distinct pattern of mediational results involving employment insecurity. Although organizational downsizing was indirectly and adversely related to 10 of the 16 outcomes via employment insecurity, the direction of the indirect association of downsizing to turnover intentions was negative rather than positive. In other words, in the face of downsizing and its negative influence on work conditions and subsequent outcomes, individuals who were concerned about not finding another job if they lost their present job reported lower levels of intentions to quit their job. Because of their perceived lack of external employment options, these individuals might feel trapped in a toxic work environment, thereby resulting in several deleterious outcomes, as the present study demonstrates.

It should be noted, however, that this last finding may be affected by the macroeconomic context in which downsizing is embedded. Feeling trapped in an undesirable work environment may be more likely when downsizing occurs during periods of economic decline than when it occurs during periods of economic growth. The present study was conducted during the Great Recession and its aftermath in the U.S., which was associated with high rates of long-term unemployment that lasted many years [40]. In general, the transition from the traditional recession to the modern or structural recession in the early 1990s brought with it a higher likelihood of permanent job loss and protracted periods of jobless recoveries [57]. Given these circumstances, it is understandable that downsizing survivors might feel that they are unable to escape poor work conditions by leaving their job during an economic decline.

### 7.4. Organizational and Public Health Implications

Despite decades of research showing that in the intermediate and long term, organizational downsizing often fails to deliver the anticipated financial and performance benefits, and may result in further financial and performance declines, it has become a staple of organizational strategy [2,3,58]. This lack of benefit has been attributed to the potential negative, though often less visible, impact of downsizing on work conditions and employees [3,58]. The present results support this contention. To minimize the potential adverse organizational outcomes of downsizing, firms can consider alternatives to layoffs, such as organizational and job redesign, as well as broad cultural change, to increase the likelihood of improved organizational performance [2,3,58]. Companies that more successfully manage the impact of external demands (e.g., recession and investor expectations for short-term profit maximization) put people first and engage them in the process of change; in other words, these companies treat employees as resources rather than costs to be eliminated [2,3,59].

If alternatives are not feasible, the present results suggest that the implementation of downsizing needs to carefully consider its potentially broad negative impact on the work environment and survivors. However, research shows that organizational downsizing is often implemented quickly with little planning and is often the result of mimicking what other organizations are doing without a critical assessment of its utility [3,58]. Unfortunately, our findings support previous qualitative observations, which were based on discussions with management about the reasons for and process used to implement layoffs, that downsizing may be implemented all too frequently with little consideration of its consequences on surviving employees [3,58].

Therefore, to better serve survivors experiencing stress, employee assistance program staff and mental health professionals need to understand the potentially broad, negative impact on survivors. Also, in the absence of direct organizational and job redesign efforts, several strategies for intervening at the level of the employee may exist. For example, there is growing evidence that training to increase psychological resilience may help minimize adverse personal outcomes in the face of undesirable work conditions [60]. Although resilience training is a reactive method of dealing with poor work conditions, a more proactive strategy that has received growing attention is job crafting [61,62]. Job crafting represents employees proactively “shaping the task boundaries of the job (either physically or cognitively), the relational boundaries of the job, or both” (p. 179) [61]. Such active job crafting may alter the nature of work in ways that reduce exposure to adverse working conditions. Recent research has begun to show that interventions to teach and stimulate job crafting may be effective [63]. Nonetheless, despite the early promise of interventions to develop psychological resilience and job crafting skills, more attention needs to focus on the development and evaluation of such interventions, especially in terms of long-term effectiveness and under various macroeconomic conditions.

### 7.5. Study Limitations

The present study utilized a large probability sample of U.S. workers, which provides (a) more variation in the variables, (b) adequate statistical power to detect hypothesized effects, and (c) more accurate estimates of population parameters [64,65,66]. Nonetheless, two study limitations should be considered. The first limitation was that because the variables were assessed at the same time, the possibility of reverse or bidirectional associations cannot be ruled out. Nonetheless, associations between organizational downsizing with both the nine work conditions and 16 employee outcomes may be less likely to be the result of reverse causation. When reporting the occurrence of downsizing, participants were reporting on an objective condition that was more likely driven by the macroeconomic conditions at the time of data collection. The second limitation was that the variables were all obtained from self-reports. Although it is typically assumed that common method variance (CMV) can inflate observed associations relative to the true population associations, CMV can lead to deflated associations as well [67,68]. To minimize processes that lead to CMV, such as consistency biases, demand characteristics, and social desirability biases, the design of this study incorporated several procedural remedies to minimize the likelihood of CMV [69]. These remedies included maintaining anonymity regarding the participants’ place of employment; interviewer training to build rapport with participants to enhance honest reporting; selection and development of items and response anchors to minimize the cognitive demands of the survey; and separation of the present measures across sections of a larger questionnaire to minimize response consistency and stylistic and careless responding, which was enhanced by the interviewer-administered telephone survey that makes prior responses physically unavailable and less likely to be available in short-term memory.

## 8. Conclusions

This study shows that the adverse effects of organizational downsizing go beyond those who lose jobs. Employees who survive a downsizing experience a wide range of harmful secondary effects (i.e., poor work conditions), which are associated with a wide variety of adverse outcomes. Downsizing was adversely related to nine of the 12 work conditions, which represented work role conditions, interpersonal relationships, rewards, and security. Downsizing also was adversely related to all 16 employee outcomes, which represented the inability to detach from work, energetic resource depletion, negative affect, positive affect, health, and work attitudes. Finally, the nine work conditions collectively mediated the association of downsizing to all 16 employee outcomes, representing both broad and narrow mediational processes. The nine mediating work conditions represent potential targets for multicomponent workplace interventions aimed at mitigating the broad set of harmful outcomes, though the specific work conditions to target in interventions may depend on the class of outcomes to be addressed by the intervention.

These adverse work conditions and outcomes occur in a context where employers must rely more heavily on a smaller group of surviving employees. Therefore, organizational leaders, unions, and public health researchers need to understand the broad adverse impact of downsizing on survivors and develop both top-down (e.g., organization-driven job resign) and bottom-up (e.g., employee job crafting) approaches to minimize both the need for layoffs and their impact on surviving employees.

## Figures and Tables

**Table 1:** Sample characteristics.

Variable	Unexposed to Downsizing Unweighted *n* = 1475 Weighted Percentage = 63.4%		Downsizing Survivors Unweighted *n* = 822 Weighted Percentage = 36.6%		Total Sample Unweighted *n* = 2297	
	Unweighted *n*	Weighted Percentage or Mean	Unweighted *n*	Weighted Percentage or Mean	Unweighted *n*	Weighted Percentage or Mean
Gender ***						
Male	543	48.5%	372	58.5%	1382	52.2%
Female	932	51.5%	450	41.5%	915	47.8%
Race						
White	1199	70.1%	651	65.5%	1850	68.4%
Minority	276	29.9%	171	34.5%	447	31.6%
Age	1475	40.0	822	40.6	2297	40.2
Education *	1475	5.8	822	6.0	2297	5.9
Family Income (median) ***	1475	60,000	822	75,000	2297	65,000
U.S. Census Division^*^						
New England	101	4.6%	54	5.1%	155	4.8%
Middle Atlantic	247	14.6%	131	12.9%	378	14.0%
East North Central	294	15.6%	153	14.4%	447	15.2%
West North Central	141	7.7%	74	6.6%	215	7.3%
South Atlantic	247	21.6%	131	19.5%	378	20.8%
East South Central	77	7.0%	32	5.7%	109	6.5%
West South Central	120	9.2%	43	7.7%	163	8.6%
Mountain	90	6.3%	56	6.2%	146	6.2%
Pacific	158	13.4%	148	22.1%	306	16.6%
Occupations ***						
Management/business/financial	176	11.1%	155	17.3%	331	13.4%
Professional	504	30.6%	321	35.1%	825	32.2%
Service	237	19.5%	50	7.0%	287	14.9%
Sales	120	8.1%	51	7.5%	171	7.9%
Office/administrative support	238	14.4%	120	14.3%	358	14.4%
Construction/extraction/farming/	26	2.3%	22	4.3%	48	3.0%
fishing/forestry						
Installation/maintenance/repair	48	4.1%	21	2.9%	69	3.7%
Production	51	3.7%	37	3.7%	88	3.7%
Transportation/material moving	75	6.2%	45	7.9%	120	6.8%
Industry ***						
Agriculture/forestry/mining/construction	35	2.9%	40	6.9%	75	4.3%
Manufacturing	108	7.7%	132	14.2%	240	10.1%
Trade	186	12.3%	74	12.3%	260	12.3%
Information sector	27	1.5%	41	5.4%	68	2.9%
Financial/real estate/management companies	96	6.2%	67	8.2%	163	6.9%d
Professional/scientific/technical services	86	5.7%	63	7.9%	149	6.5%
Education services	232	13.9%	149	14.5%	381	14.1%
Health services	303	17.3%	113	10.7%	416	14.9%
Leisure/hospitality	116	11.4%	31	6.6%	147	9.6%
Administrative/support/other services	84	6.9%	46	5.5%	130	6.4%
Government/utilities	146	10.4%	36	3.6%	182	7.9%
Transportation	56	3.8%	30	4.3%	86	4.0%
Employer has more than one work location ***						
No	530	35.5%	181	24.3%	711	31.4%
Yes	945	64.5%	641	75.7%	1586	68.6%
Number of employees at workplace ***	1475	3.8	822	4.8	2297	4.2
Number of total employees ***	1475	5.9	822	7.2	2297	6.4
Job Tenure (years)	1475	5.3	822	5.6	2297	5.4
Number of Weekly Work Hours ***	1475	39.6	822	42.9	2297	40.8
Seasonal Job						
No	1395	92.8%	793	95.2%	2188	93.7%
Yes	80	7.2%	29	4.8%	109	6.3%
Precarious employment						
No	1284	86.3%	704	83.3%	1988	85.2%
Yes	191	13.7%	118	16.7%	309	14.8%
Union Member **						
No	1224	84.6%	627	79.0%	1851	82.5%
Yes	251	15.4%	195	21.0%	446	17.5%
Calendar quarter ***	1475	4.5	822	4.1	2297	4.4

Note: Weighted means or percentages differed across Studies 1 and 2 at * *p* < 0.05, ** *p* < 0.01, *** *p* < 0.001. See the Measures section for a description of the variables.

**Table 2:** Unstandardized path coefficients relating organizational downsizing to work conditions (weighted).

Work Conditions	*b* (SE)	Work Conditions	*b* (SE)
**WORK ROLE**		**REWARDS**	
Work demands	0.178 (0.065) **	Distributive justice	−0.272 (0.057) ***
Role conflict	0.193 (0.057) ***	Promotion opportunities	−0.297 (0.059) ***
Role ambiguity	0.058 (0.036)		
Work autonomy	0.037 (0.040)		
**INTERPERSONAL**		**SECURITY**	
Supervisor aggression	0.098 (0.043) *	Job insecurity	0.450 (0.054) ***
Coworker aggression	0.066 (0.051)	Employment insecurity	0.234 (0.051) ***
Friendship formation	−0.172 (0.050) ***		
Dysfunctional leadership	0.180 (0.048) ***		

Note: *n* = 2297. All coefficients are adjusted for the covariates shown in Table 1. Robust nonparametric bootstrap standard errors are reported. * *p* ≤ 0.05, ** *p* ≤ 0.01, *** *p* ≤ 0.001.

**Table 3:** Unstandardized total effects, total indirect effects, and direct effects of organizational downsizing on employee outcomes (weighted).

Employee Outcomes	Total Effect *b* (SE)	Total Indirect Effect *b* (SE)	Direct Effect *b* (SE)
**INABILITY TO DETACH**			
Negative work rumination	0.127 (0.044) **	0.084 (0.021) ***	0.043 (0.042)
Inability to unwind	0.187 (0.074) *	0.156 (0.036) ***	0.031 (0.073)
**ENERGY DEPLETION**			
Physical work fatigue	0.070 (0.070)	0.123 (0.033) ***	−0.053 (0.068)
Mental work fatigue	0.126 (0.070)	0.150 (0.036) ***	−0.024 (0.071)
Emotional work fatigue	0.124 (0.063) *	0.187 (0.035) ***	−0.063 (0.061)
**NEGATIVE AFFECT**			
Depression	0.115 (0.039) **	0.078 (0.019) ***	0.037 (0.041)
Anxiety	0.121 (0.038) **	0.070 (0.018) ***	0.050 (0.039)
Anger	0.131 (0.038) ***	0.038 (0.017) *	0.093 (0.039) *
**POSITIVE AFFECT**			
Happiness	−0.087 (0.028) **	−0.055 (0.012) ***	−0.032 (0.028)
Confidence	−0.080 (0.030) **	−0.048 (0.013) ***	−0.032 (0.029)
Vigor	−0.087 (0.031) **	−0.056 (0.013) ***	−0.032 (0.031)
**HEALTH**			
Physical	−0.064 (0.051)	−0.059 (0.018) ***	−0.006 (0.051)
Mental	−0.125 (0.054) *	−0.116 (0.024) ***	−0.009 (0.056)
**WORK ATTITUDES**			
Job satisfaction	−0.195 (0.051) ***	−0.170 (0.033) ***	−0.026 (0.042)
Organizational commitment	−0.258 (0.055) ***	−0.198 (0.037) ***	−0.060 (0.048)
Turnover intentions	0.203 (0.062) ***	0.211 (0.037) ***	−0.009 (0.054)

Note: *n* = 2297. All coefficients are adjusted for the covariates shown in Table 1. Robust nonparametric bootstrap standard errors are reported. * *p* ≤ 0.05, ** *p* ≤ 0.01, *** *p* ≤ 0.001.

**Table 4:** Statistically significant mediators of the associations of organizational downsizing to employee outcomes.

Employee Outcomes/ Mediators	Indirect Effect *b* (95% BC CI)	Employee Outcomes/ Mediators	Indirect Effect *b* (95% BC CI)
**INABILITY TO DETACH**		**POSITIVE AFFECT**	
**Negative work rumination**		**Happiness**	
Work demands	0.019 (0.006, 0.040)	Friendship formation	−0.010 (−0.020, −0.004)
Dysfunctional leadership	0.022 (0.008, 0.047)	Dysfunctional leadership	−0.008 (−0.020, −0.001)
Distributive justice	0.018 (0.006, 0.033)	Distributive justice	−0.008 (−0.018, −0.001)
**Inability to unwind**		Employment insecurity	−0.011 (−0.021, −0.005)
Work demands	0.045 (0.015, 0.086)	**Confidence**	
Role conflict	0.023 (0.007, 0.051)	Friendship formation	−0.006 (−0.016, −0.001)
Dysfunctional leadership	0.025 (0.007, 0.054)	Distributive justice	−0.010 (−0.021, −0.003)
Job insecurity	0.052 (0.013, 0.096)	Employment insecurity	−0.017 (−0.028, −0.008)
		**Vigor**	
**ENERGY DEPLETION**		Friendship formation	−0.008 (−0.019, −0.002)
**Physical work fatigue**		Distributive justice	−0.013 (−0.026, −0.004)
Work demands	0.045 (0.015, 0.087)	Employment insecurity	−0.016 (−0.028, −0.007)
Supervisor aggression	0.020 (0.005, 0.048)		
Employment insecurity	0.031 (0.013, 0.056)	**HEALTH**	
**Mental work fatigue**		**Physical health**	
Work demands	0.052 (0.018, 0.098)	Friendship formation	−0.009 (−0.024, −0.001)
Supervisor aggression	0.018 (0.003, 0.046)	Distributive justice	−0.021 (−0.043, −0.007)
Job insecurity	0.040 (0.003, 0.082)	**Mental health**	
Employment insecurity	0.020 (0.005, 0.043)	Supervisor aggression	−0.012 (−0.031, −0.002)
**Emotional work fatigue**		Distributive justice	−0.024 (−0.046, −0.010)
Work demands	0.035 (0.012, 0.070)	Job insecurity	−0.043 (−0.080, −0.011)
Role conflict	0.021 (0.008, 0.042)	Employment insecurity	−0.026 (−0.047, −0.012)
Supervisor aggression	0.012 (0.001, 0.031)		
Dysfunctional leadership	0.019 (0.003, 0.044)	**WORK ATTITUDES**	
Job insecurity	0.057 (0.024, 0.100)	**Job satisfaction**	
Employment insecurity	0.022 (0.007, 0.043)	Friendship formation	−0.024 (−0.044, −0.010)
		Dysfunctional leadership	−0.033 (−0.062, −0.014)
**NEGATIVE AFFECT**		Distributive justice	−0.036 (−0.060, −0.020)
**Depression**		Promotion opportunity	−0.024 (−0.043, −0.011)
Work demands	0.007 (0.001, 0.019)	Job insecurity	−0.026 (−0.051, −0.001)
Friendship formation	0.009 (0.002, 0.021)	**Organizational commitment**	
Employment insecurity	0.016 (0.006, 0.030)	Friendship formation	−0.042 (−0.072, −0.019)
**Anxiety**		Dysfunctional leadership	−0.039 (−0.070, −0.019)
Work demands	0.009 (0.002, 0.023)	Distributive justice	−0.047 (−0.074, −0.026)
Friendship formation	0.008 (0.001, 0.020)	Promotion opportunity	−0.044 (−0.072, −0.025)
Distributive justice	0.010 (0.001, 0.024)	**Turnover intentions**	
Employment insecurity	0.023 (0.012, 0.041)	Role conflict	0.015 (0.005, 0.033)
**Anger**		Friendship formation	0.021 (0.008, 0.043)
Work demands	0.010 (0.003, 0.024)	Dysfunctional leadership	0.038 (0.017, 0.070)
Friendship formation	0.010 (0.002, 0.024)	Distributive justice	0.042 (0.022, 0.069)
Dysfunctional leadership	0.011 (0.001, 0.028)	Promotion opportunity	0.043 (0.023, 0.071)
Distributive justice	0.014 (0.003, 0.030)	Job insecurity	0.046 (0.018, 0.0081)
		Employment insecurity	−0.018 (−0.034, −0.006)

Note: *n* = 2297. BC CI = Bias corrected bootstrap confidence intervals based on 5000 bootstrapped samples. All coefficients are adjusted for the covariates shown in Table 1.

**Table 5:** Mapping statistically significant mediating work conditions to employee outcome groups and specific outcomes.

Mediating Work Conditions	Outcome Group	Specific Outcomes
**WORK ROLE**		
Work demands	Inability to detach	Negative work rumination (+); Inability to unwind (+)
	Energy depletion	Physical work fatigue (+); Mental work fatigue (+); Emotional work fatigue (+)
	Negative affect	Depression (+); Anxiety (+); Anger (+)
Role conflict	Inability to detach	Inability to unwind (+)
	Energy depletion	Emotional work fatigue (+)
	Work attitudes	Turnover intentions (+)
**INTERPERSONAL**		
Supervisor aggression	Energy depletion	Physical work fatigue (+); Mental work fatigue (+); Emotional work fatigue (+)
	Health	Mental health (−)
Friendship formation	Negative affect	Depression (+); Anxiety (+); Anger (+)
	Positive affect	Happiness (−); Confidence (−); Vigor (−)
	Health	Physical health (−)
	Work attitudes	Job satisfaction (−); Organizational commitment (−); Turnover intentions (+)
Dysfunctional leadership	Inability to detach	Negative work rumination (+); Inability to unwind (+)
	Energy depletion	Emotional work fatigue (+)
	Negative affect	Anger (+)
	Positive affect	Happiness (−)
	Work attitudes	Job satisfaction (−); Organizational commitment (−); Turnover intentions (+)
**REWARDS**		
Distributive justice	Inability to detach	Negative work rumination (+)
	Negative affect	Anxiety (+); Anger (+)
	Positive affect	Happiness (−); Confidence (−); Vigor (−)
	Health	Physical health (−); Mental health (−)
	Work attitudes	Job satisfaction (−); Organizational commitment (−); Turnover intentions (+)
Promotion opportunities	Work attitudes	Job satisfaction (−); Organizational commitment (−); Turnover intentions (+)
**SECURITY**		
Job insecurity	Inability to detach	Inability to unwind (+)
	Energy depletion	Mental work fatigue (+); Emotional work fatigue (+)
	Health	Mental health (−)
Employment insecurity	Work attitudes	Job satisfaction (−); Turnover intentions (+)
	Energy depletion	Physical work fatigue (+); Mental work fatigue (+); Emotional work fatigue (+)
	Negative affect	Depression (+); Anxiety (+)
	Positive affect	Happiness (−); Confidence (−); Vigor (−)
	Health	Mental health (−)
	Work attitudes	Turnover intentions (−)

Note: Of the 12 work conditions, only role ambiguity, work autonomy, and coworker aggression failed to act as a mediating variable in the associations of organizational downsizing to the employee outcomes. The plus and minus signs represent the direction of the indirect effect of organizational downsizing to a specific employee outcome (column 3) via a specific work condition (column 1).

## Supplementary Materials

The following are available online at https://www.mdpi.com/1660-4601/17/3/719/s1, Appendix S1: Descriptive statistics and correlations, and Appendix S2: Standardized factor loadings.
