# Supplementary material for: Organizational Downsizing, Work Conditions, and Employee Outcomes: Identifying Targets for Workplace Intervention among Survivors
Source: Int J Environ Res Public Health. 2020 Jan 22;17(3):719. doi: 10.3390/ijerph17030719 (PMC7037986; doi:10.3390/ijerph17030719)
Supplement: Supplementary file 1 [file ijerph-17-00719-s001.zip › Appendix S1.pdf]

Appendix S1

This appendix contains a key linking variable names with construct names, as well as means, standard deviations, and correlations for all 70 variables used in the structural model. See the Measures section in the article for the scoring of all variables. For reported correlations, a correlation with an absolute value ≥ .041 is significant at *p* <.05.

Variable to Construct Key

Variable name	Construct name	Variable name	Construct Name
LAYOFF	Organizational downsizing	**U.S. Census region**	**U.S. Census region**
WRKDMND	Work demands	MA	Middle Atlantic
ROLECONF	Role conflict	ENC	East North Central
ROLEAMB	Role ambiguity	WNC	West North Central
WORKAUT	Work autonomy	SA	South Atlantic
COWAGGR2	Coworker aggression	ESC	East South Central
SUPAGGR2	Supervisor aggression	WSC	West South Central
FRNDFRM2	Friendship formation	MNT	Mountain
POORLDR	Dysfunctional leadership	PAC	Pacific
ORGJUST	Distributive justice	**Occupation categories**	**Occupation categories**
PROMO2	Promotion opportunity	SOC2NN2	Professional
JOBINSEC	Job insecurity	SOC2NN3	Service
EMPINSEC	Employment insecurity	SOC2NN4	Sales
NEGRUM	Negative work rumination	SOC2NN5	Office/administrative support
IUFREQ	Inability to unwind	SOC2NN7	Construction/extraction/farming/fishing/forestry
PFAT	Physical work fatigue	SOC2NN8	Installation/maintenance/repair
MFAT	Mental work fatigue	SOC2NN9	Production
EFAT	Emotional work fatigue	SOC2NN10	Transportation/material moving
DEP	Depression	**Industry categories**	**Industry categories**
ANX	Anxiety	NAICSA2	Manufacturing
ANG	Anger	NAICSA3	Trade
HAP	Happiness	NAICSA4	Information sector
CONF	Confidence	NAICSA5	Financial/real estate/management companies
VIG	Vigor	NAICSA6	Professional/scientific/technical services
PHLTHO	Physical health	NAICSA7	Education services
MHLTHO	Mental health	NAICSA8	Health services
JOBSAT	Job satisfaction	NAICSA9	Leisure/hospitality
ORGCOMIT	Organizational commitment	NAICSA10	Administrative/support/other services
TURNOVER	Turnover intentions	NAICSA11	Government/utilities
GENDERM	Gender	NAICSA12	Transportation
RACE2	Race	JTYEAR	Job tenure
AGE	Age	NWHRS60	Number of weekly work hours
EDUC	Education	SEASONAL	Seasonal job
INCOME10	Total family income	PRECAREM	Precarious employment
NWRKLOC	Employer has more than one work location	UNION	Union member
EMPWLOC	Number of employees at work location	QUARTER	Calendar quarter of interview
EMPWTOT	Total number of employees in the organization		

Means / Standard Deviations

LAYOFF	WRKDMND	ROLECONF	ROLEAMB	WORKAUT
0.366 / 0.482	2.453 / 1.088	2.599 / 0.964	1.504 / 0.601	3.365 / 0.657

COWAGGR2	SUPAGGR2	FRNDFRM2	POORLDR	ORGJUST
0.462 / 0.747	0.466 / 0.727	3.209 / 0.804	1.864 / 0.777	2.813 / 0.981

PROMO2	JOBINSEC	EMPINSEC	NEGRUM	IUFREQ
2.356 / 1.001	1.782 / 0.872	2.177 / 0.913	1.696 / 0.782	1.846 / 1.237

PFAT	MFAT	EFAT	DEP	ANX
1.845 / 1.153	1.746 / 1.159	1.196 / 1.094	1.316 / 0.644	1.803 / 0.652

ANG	HAP	CONF	VIG	PHLTHO
1.249 / 0.640	2.692 / 0.444	2.546 / 0.489	2.540 / 0.527	3.625 / 0.838

MHLTHO	JOBSAT	ORGCOMIT	TURNOVER	GENDERM
3.801 / 0.871	3.404 / 0.786	2.918 / 0.971	1.839 / 0.966	0.522 / 0.500

RACE2	AGE	EDUC	INCOME10	MA
0.316 / 0.465	40.248 / 12.659	5.860 / 2.225	8.112 / 12.211	0.140 / 0.346

ENC	WNC	SA	ESC	WSC
0.152 / 0.359	0.073 / 0.261	0.208 / 0.406	0.065 / 0.247	0.086 / 0.279
MNT	PAC	SOC2NN2	SOC2NN3	SOC2NN4
0.062 / 0.243	0.166 / 0.371	0.322 / 0.467	0.149 / 0.356	0.079 / 0.270
SOC2NN5	SOC2NN7	SOC2NN8	SOC2NN9	SOC2NN10
0.144 / 0.351	0.030 / 0.170	0.037 / 0.187	0.037 / 0.190	0.068 / 0.253
NAICSA2	NAICSA3	NAICSA4	NAICSA5	NAICSA6
0.101 / 0.302	0.123 / 0.329	0.029 / 0.167	0.069 / 0.255	0.065 / 0.247
NAICSA7	NAICSA8	NAICSA9	NAICSA10	NAICSA11
0.141 / 0.348	0.149 / 0.356	0.096 / 0.295	0.064 / 0.245	0.079 / 0.270
NAICSA12	NWRKLOC	EMPWLOC	EMPWTOT	JTYEAR
0.040 / 0.195	0.686 / 0.464	4.166 / 2.710	6.387 / 3.448	5.407 / 6.314
NWHRS60	SEASONAL	PRECAREM	UNION	QUARTER
40.838 / 11.483	0.063 / 0.243	0.148 / 0.355	0.175 / 0.379	4.366 / 2.321

Correlations

	WRKDMND	ROLECONF	ROLEAMB	WORKAUT	COWAGGR2
WRKDMND	1.000				
ROLECONF	0.347	1.000			
ROLEAMB	0.203	0.310	1.000		
WORKAUT	-0.050	-0.016	-0.203	1.000	
COWAGGR2	0.199	0.249	0.165	-0.133	1.000
SUPAGGR2	0.273	0.329	0.311	-0.250	0.431
FRNDFRM2	-0.056	-0.060	-0.165	0.118	-0.113
POORLDR	0.337	0.424	0.418	-0.231	0.333
ORGJUST	-0.213	-0.265	-0.325	0.225	-0.164
PROMO2	-0.138	-0.100	-0.179	0.082	-0.088
JOBINSEC	0.113	0.135	0.250	-0.223	0.093
EMPINSEC	0.060	0.034	0.159	-0.200	0.074
NEGRUM	0.322	0.210	0.228	-0.007	0.149
IUFREQ	0.365	0.245	0.147	-0.045	0.164
PFAT	0.336	0.182	0.119	-0.155	0.187
MFAT	0.391	0.215	0.184	-0.067	0.149
EFAT	0.338	0.272	0.202	-0.090	0.208
DEP	0.163	0.113	0.156	-0.110	0.163
ANX	0.169	0.129	0.136	-0.112	0.115
ANG	0.173	0.100	0.105	-0.043	0.162
HAP	-0.132	-0.118	-0.144	0.101	-0.158
CONF	-0.091	-0.066	-0.182	0.150	-0.106
VIG	-0.081	-0.099	-0.148	0.110	-0.127
PHLTHO	-0.060	-0.094	-0.098	0.080	-0.063
MHLTHO	-0.117	-0.139	-0.156	0.118	-0.156
JOBSAT	-0.219	-0.290	-0.357	0.304	-0.293

	WRKDMND	ROLECONF	ROLEAMB	WORKAUT	COWAGGR2
ORGCOMIT	-0.137	-0.239	-0.278	0.277	-0.235
TURNOVER	0.185	0.301	0.302	-0.181	0.240
LAYOFF	0.136	0.125	0.085	0.047	0.051
GENDERM	0.003	0.047	0.082	0.037	0.057
RACE2	-0.036	0.046	-0.033	-0.073	-0.009
AGE	0.071	-0.039	0.010	0.142	-0.084
EDUC	0.136	-0.030	0.153	0.115	-0.101
INCOME10	0.080	0.018	0.090	0.123	0.042
MA	0.005	-0.004	-0.016	-0.015	-0.015
ENC	0.017	-0.006	-0.002	-0.029	0.010
WNC	0.005	0.008	0.039	-0.040	0.000
SA	-0.037	-0.050	-0.022	-0.011	-0.060
ESC	-0.044	0.018	-0.021	-0.020	-0.029
WSC	-0.028	-0.016	-0.034	0.041	0.079
MNT	0.016	0.042	0.031	0.045	0.037
PAC	0.063	0.035	0.027	0.029	0.008
SOC2NN2	0.115	-0.041	0.067	0.053	-0.084
SOC2NN3	-0.093	0.013	-0.028	-0.096	0.041
SOC2NN4	0.001	0.049	-0.058	-0.012	0.049
SOC2NN5	-0.062	-0.009	-0.016	-0.053	-0.024
SOC2NN7	-0.003	0.004	-0.015	0.010	-0.017
SOC2NN8	-0.043	-0.043	-0.019	0.022	0.030
SOC2NN9	-0.012	-0.016	-0.039	-0.030	0.054
SOC2NN10	-0.041	0.026	-0.079	-0.103	0.078
NAICSA2	0.013	0.037	0.058	0.009	0.061
NAICSA3	0.016	0.086	-0.045	-0.044	0.114
NAICSA4	0.009	0.017	0.039	0.050	-0.047

	WRKDMND	ROLECONF	ROLEAMB	WORKAUT	COWAGGR2
NAICSA5	-0.003	-0.033	0.001	0.059	-0.034
NAICSA6	0.060	0.008	0.115	0.023	-0.062
NAICSA7	0.040	-0.066	-0.033	0.027	-0.076
NAICSA8	0.016	-0.008	-0.025	0.016	-0.023
NAICSA9	-0.065	0.021	-0.057	-0.096	0.039
NAICSA10	-0.044	-0.019	-0.006	0.035	-0.032
NAICSA11	-0.055	-0.044	0.002	0.028	0.008
NAICSA12	-0.018	-0.032	-0.030	-0.128	0.021
NWRKLOC	0.072	0.050	0.008	0.001	0.046
EMPWLOC	0.066	0.062	0.049	-0.008	0.046
EMPWTOT	0.111	0.069	0.047	-0.037	0.075
JTYEAR	0.074	-0.031	-0.060	0.083	-0.027
NWHRS60	0.248	0.133	0.083	0.168	0.086
SEASONAL	-0.001	-0.006	-0.049	-0.057	-0.018
PRECAREM	-0.010	-0.008	-0.018	-0.026	0.019
UNION	0.080	-0.037	-0.062	-0.089	0.030
QUARTER	-0.028	-0.006	-0.032	-0.026	-0.014

	SUPAGGR2	FRNDFRM2	POORLDR	ORGJUST	PROMO2
SUPAGGR2	1.000				
FRNDFRM2	-0.102	1.000			
POORLDR	0.492	-0.200	1.000		
ORGJUST	-0.277	0.160	-0.411	1.000	
PROMO2	-0.177	0.094	-0.276	0.387	1.000
JOBINSEC	0.206	-0.178	0.246	-0.282	-0.233
EMPINSEC	0.072	-0.156	0.102	-0.170	-0.179
NEGRUM	0.189	-0.040	0.264	-0.222	-0.130
IUFREQ	0.201	-0.016	0.257	-0.153	-0.107
PFAT	0.273	-0.069	0.256	-0.191	-0.116
MFAT	0.246	-0.009	0.217	-0.175	-0.096
EFAT	0.268	-0.102	0.297	-0.206	-0.131
DEP	0.187	-0.110	0.193	-0.178	-0.127
ANX	0.139	-0.094	0.164	-0.165	-0.097
ANG	0.132	-0.094	0.171	-0.147	-0.048
HAP	-0.155	0.180	-0.207	0.184	0.126
CONF	-0.111	0.122	-0.152	0.183	0.146
VIG	-0.141	0.135	-0.168	0.194	0.125
PHLTHO	-0.116	0.078	-0.112	0.145	0.057
MHLTHO	-0.216	0.079	-0.165	0.197	0.091
JOBSAT	-0.388	0.305	-0.484	0.424	0.264
ORGCOMIT	-0.323	0.340	-0.430	0.386	0.246
TURNOVER	0.349	-0.237	0.434	-0.372	-0.272
LAYOFF	0.043	-0.112	0.115	-0.131	-0.095
GENDERM	0.040	-0.094	0.040	0.043	0.094
RACE2	0.023	-0.086	0.047	-0.051	0.065
AGE	-0.112	0.029	-0.048	-0.016	-0.173

	SUPAGGR2	FRNDFRM2	POORLDR	ORGJUST	PROMO2
EDUC	-0.098	0.009	-0.049	-0.059	-0.026
INCOME10	0.006	0.015	0.004	0.037	0.018
MA	0.017	0.052	-0.024	-0.040	-0.013
ENC	0.005	-0.029	0.002	0.022	-0.005
WNC	0.016	0.057	0.006	-0.026	-0.022
SA	0.011	-0.019	-0.018	-0.024	0.014
ESC	-0.023	-0.003	-0.032	-0.023	0.002
WSC	-0.047	-0.026	0.037	0.042	-0.003
MNT	0.004	0.006	0.049	-0.011	-0.021
PAC	0.011	-0.028	0.012	0.059	0.020
SOC2NN2	-0.140	0.084	-0.052	-0.046	-0.061
SOC2NN3	0.103	-0.056	0.031	0.017	0.033
SOC2NN4	0.091	0.027	0.011	0.083	0.069
SOC2NN5	0.011	-0.026	-0.003	-0.035	-0.047
SOC2NN7	0.010	0.013	0.017	0.041	-0.006
SOC2NN8	0.020	-0.028	-0.019	0.006	0.005
SOC2NN9	-0.008	0.025	-0.002	0.034	0.047
SOC2NN10	0.047	-0.064	0.102	-0.092	-0.051
NAICSA2	-0.031	0.018	-0.037	0.032	0.039
NAICSA3	0.031	-0.077	0.094	-0.016	0.023
NAICSA4	-0.029	-0.052	-0.024	-0.023	0.021
NAICSA5	0.008	-0.013	-0.044	0.030	0.039
NAICSA6	-0.018	-0.016	-0.014	0.018	0.034
NAICSA7	-0.093	0.078	-0.028	-0.047	-0.096
NAICSA8	-0.013	0.052	-0.009	-0.020	-0.030
NAICSA9	0.076	0.041	0.023	0.087	0.057
NAICSA10	-0.002	-0.095	-0.001	0.005	-0.060

	SUPAGGR2	FRNDFRM2	POORLDR	ORGJUST	PROMO2
	________	________	________	________	________
NAICSA11	-0.014	0.015	-0.057	-0.011	0.046
NAICSA12	0.085	-0.034	0.072	-0.095	-0.042
NWRKLOC	-0.026	-0.025	0.010	-0.060	0.099
EMPWLOC	-0.036	-0.052	0.008	-0.066	0.091
EMPWTOT	-0.001	-0.044	0.032	-0.100	0.135
JTYEAR	-0.068	0.083	0.004	-0.017	-0.177
NWHRS60	-0.009	0.066	0.102	-0.067	0.038
SEASONAL	-0.009	-0.005	-0.011	0.017	0.010
PRECAREM	0.016	-0.093	-0.022	-0.038	-0.054
UNION	0.004	0.024	0.042	-0.074	-0.014
QUARTER	0.019	0.043	-0.032	0.025	0.037

	JOBINSEC	EMPINSEC	NEGRUM	IUFREQ	PFAT
JOBINSEC	1.000				
EMPINSEC	0.366	1.000			
NEGRUM	0.070	0.077	1.000		
IUFREQ	0.127	0.050	0.481	1.000	
PFAT	0.123	0.140	0.264	0.399	1.000
MFAT	0.146	0.110	0.393	0.456	0.612
EFAT	0.199	0.139	0.380	0.460	0.541
DEP	0.168	0.155	0.333	0.247	0.299
ANX	0.142	0.178	0.330	0.258	0.240
ANG	0.046	0.054	0.317	0.203	0.193
HAP	-0.170	-0.166	-0.197	-0.167	-0.224
CONF	-0.140	-0.194	-0.174	-0.085	-0.165
VIG	-0.146	-0.174	-0.187	-0.159	-0.254
PHLTHO	-0.109	-0.101	-0.134	-0.155	-0.278
MHLTHO	-0.194	-0.181	-0.266	-0.221	-0.293
JOBSAT	-0.301	-0.159	-0.217	-0.184	-0.192
ORGCOMIT	-0.255	-0.085	-0.099	-0.074	-0.138
TURNOVER	0.267	0.050	0.168	0.168	0.185
LAYOFF	0.250	0.138	0.092	0.083	0.029
GENDERM	0.014	-0.006	-0.123	-0.080	-0.142
RACE2	0.087	0.024	-0.160	-0.040	0.045
AGE	0.020	0.073	0.119	0.041	-0.030
EDUC	-0.046	-0.038	0.235	0.113	-0.045
INCOME10	0.008	0.000	0.142	0.090	0.011
MA	0.003	0.045	0.018	0.005	-0.011
ENC	0.007	0.064	0.020	0.009	-0.004
WNC	-0.018	-0.018	0.019	-0.009	0.001

	JOBINSEC	EMPINSEC	NEGRUM	IUFREQ	PFAT
SA	-0.013	-0.081	-0.026	-0.022	-0.017
ESC	-0.002	-0.017	-0.049	-0.040	-0.038
WSC	-0.073	-0.064	-0.012	0.048	0.015
MNT	0.004	-0.008	0.005	-0.022	-0.023
PAC	0.061	0.054	0.003	0.021	0.074
SOC2NN2	-0.021	-0.039	0.123	0.095	-0.019
SOC2NN3	-0.005	-0.032	-0.072	-0.038	0.037
SOC2NN4	-0.034	-0.026	-0.025	-0.017	0.018
SOC2NN5	0.034	0.048	-0.024	-0.050	0.025
SOC2NN7	0.060	0.020	-0.059	-0.027	0.054
SOC2NN8	-0.020	0.044	-0.005	-0.019	-0.011
SOC2NN9	-0.004	0.000	-0.056	-0.008	0.023
SOC2NN10	0.041	0.009	-0.112	-0.048	-0.012
NAICSA2	0.076	0.097	-0.011	0.005	-0.013
NAICSA3	0.020	-0.041	-0.075	0.019	0.036
NAICSA4	0.051	0.017	0.022	0.000	-0.037
NAICSA5	-0.018	0.000	0.028	-0.017	-0.064
NAICSA6	0.013	0.007	0.024	0.031	-0.038
NAICSA7	-0.055	-0.008	0.104	0.064	0.038
NAICSA8	-0.060	-0.086	0.067	0.009	0.024
NAICSA9	-0.020	-0.029	-0.128	-0.065	0.005
NAICSA10	0.057	-0.007	-0.060	-0.062	-0.056
NAICSA11	-0.071	0.005	0.064	0.025	-0.029
NAICSA12	0.018	0.056	-0.049	-0.051	0.044
NWRKLOC	0.012	0.024	0.028	0.002	0.012
EMPWLOC	0.004	0.097	0.059	-0.026	-0.029
EMPWTOT	-0.022	0.053	0.078	0.003	0.037

	JOBINSEC	EMPINSEC	NEGRUM	IUFREQ	PFAT
JTYEAR	-0.040	0.071	0.057	-0.017	-0.035
NWHRS60	-0.099	-0.073	0.241	0.256	0.091
SEASONAL	0.116	0.049	-0.077	-0.032	0.108
PRECAREM	0.164	0.036	-0.043	-0.023	0.078
UNION	-0.028	0.079	0.060	0.052	0.043
QUARTER	-0.035	0.020	-0.086	-0.022	-0.042

	MFAT	EFAT	DEP	ANX	ANG
MFAT	1.000				
EFAT	0.692	1.000			
DEP	0.321	0.372	1.000		
ANX	0.276	0.311	0.576	1.000	
ANG	0.229	0.273	0.536	0.414	1.000
HAP	-0.227	-0.320	-0.360	-0.200	-0.230
CONF	-0.173	-0.224	-0.279	-0.192	-0.140
VIG	-0.227	-0.284	-0.297	-0.189	-0.191
PHLTHO	-0.204	-0.204	-0.235	-0.212	-0.156
MHLTHO	-0.296	-0.353	-0.517	-0.411	-0.309
JOBSAT	-0.189	-0.298	-0.234	-0.211	-0.194
ORGCOMIT	-0.103	-0.152	-0.156	-0.130	-0.124
TURNOVER	0.163	0.240	0.209	0.155	0.151
LAYOFF	0.080	0.059	0.065	0.069	0.088
GENDERM	-0.105	-0.083	-0.168	-0.157	-0.103
RACE2	-0.015	0.018	-0.065	-0.128	-0.032
AGE	-0.007	0.025	0.013	-0.032	-0.010
EDUC	0.133	0.051	0.040	0.044	0.045
INCOME10	0.092	0.060	0.025	0.008	0.024
MA	-0.016	-0.018	-0.026	-0.001	-0.006
ENC	-0.007	0.020	0.035	0.024	-0.010
WNC	-0.012	-0.032	0.017	-0.023	-0.052
SA	0.006	-0.007	0.003	-0.005	0.065
ESC	-0.022	-0.010	-0.024	-0.001	-0.036
WSC	-0.027	0.018	-0.024	-0.029	0.018
MNT	0.013	-0.011	-0.027	-0.043	-0.026
PAC	0.056	0.036	0.031	0.044	-0.010

	MFAT	EFAT	DEP	ANX	ANG
SOC2NN2	0.069	0.000	0.030	0.045	0.034
SOC2NN3	-0.068	-0.007	-0.012	0.013	0.010
SOC2NN4	0.015	0.034	-0.013	-0.013	-0.021
SOC2NN5	0.044	0.057	0.054	0.040	-0.018
SOC2NN7	0.019	-0.017	-0.005	-0.036	0.015
SOC2NN8	-0.043	-0.053	-0.017	-0.017	0.021
SOC2NN9	-0.027	-0.038	-0.049	-0.026	-0.009
SOC2NN10	-0.116	-0.030	-0.027	-0.058	-0.053
NAICSA2	0.007	-0.011	-0.048	-0.042	-0.032
NAICSA3	-0.058	0.026	-0.024	0.007	-0.026
NAICSA4	0.011	-0.009	-0.003	0.013	-0.030
NAICSA5	-0.008	0.002	0.006	-0.018	0.020
NAICSA6	0.046	0.007	-0.006	0.008	0.022
NAICSA7	0.045	0.046	0.040	0.052	0.065
NAICSA8	0.010	-0.007	0.048	0.036	-0.002
NAICSA9	-0.040	-0.061	-0.025	-0.012	0.006
NAICSA10	-0.058	-0.014	0.007	-0.010	-0.046
NAICSA11	0.032	0.032	-0.020	-0.019	0.005
NAICSA12	-0.021	-0.013	0.014	-0.038	-0.047
NWRKLOC	0.045	0.038	-0.015	0.028	-0.009
EMPWLOC	0.024	-0.009	-0.040	-0.039	-0.006
EMPWTOT	0.061	0.039	0.002	0.013	0.022
JTYEAR	-0.020	-0.011	-0.010	-0.027	-0.031
NWHRS60	0.179	0.156	-0.001	-0.009	0.096
SEASONAL	0.007	0.007	-0.005	0.009	-0.045
PRECAREM	0.002	0.024	0.046	0.052	0.015

	MFAT	EFAT	DEP	ANX	ANG
UNION	0.031	0.001	-0.012	-0.005	0.012
QUARTER	-0.040	-0.021	-0.043	-0.022	-0.040

	HAP	CONF	VIG	PHLTHO	MHLTHO
	________	________	________	________	________
HAP	1.000				
CONF	0.579	1.000			
VIG	0.632	0.622	1.000		
PHLTHO	0.188	0.204	0.314	1.000	
MHLTHO	0.408	0.353	0.374	0.444	1.000
JOBSAT	0.315	0.271	0.265	0.124	0.260
ORGCOMIT	0.194	0.180	0.169	0.072	0.132
TURNOVER	-0.217	-0.190	-0.195	-0.100	-0.193
LAYOFF	-0.105	-0.075	-0.079	-0.038	-0.057
GENDERM	-0.079	0.010	0.009	0.056	0.057
RACE2	0.007	0.075	0.019	0.005	0.116
AGE	-0.013	0.032	-0.026	0.001	0.056
EDUC	0.028	-0.001	0.019	0.085	0.073
INCOME10	-0.024	0.024	-0.007	0.015	-0.008
MA	-0.038	-0.016	-0.044	0.010	-0.033
ENC	-0.023	-0.008	-0.013	-0.049	-0.063
WNC	-0.026	-0.037	-0.030	0.021	-0.016
SA	0.034	0.031	0.019	-0.013	0.032
ESC	0.042	-0.007	0.022	-0.011	0.039
WSC	0.011	0.016	0.019	0.118	0.059
MNT	0.004	-0.023	0.011	-0.007	0.042
PAC	0.009	0.012	0.030	-0.033	-0.027
SOC2NN2	0.063	0.002	0.042	0.032	0.040
SOC2NN3	0.011	0.035	0.018	0.001	0.009
SOC2NN4	-0.008	0.023	0.004	0.064	0.018
SOC2NN5	-0.019	-0.072	-0.068	-0.080	-0.079
SOC2NN7	-0.014	0.039	0.028	0.032	0.012

	HAP	CONF	VIG	PHLTHO	MHLTHO
SOC2NN8	-0.014	-0.029	-0.016	-0.042	-0.034
SOC2NN9	-0.001	0.033	0.036	-0.084	-0.004
SOC2NN10	-0.056	-0.021	-0.026	0.021	-0.053
NAICSA2	-0.016	0.008	-0.020	-0.074	-0.009
NAICSA3	-0.035	0.001	0.005	0.013	-0.020
NAICSA4	-0.012	-0.043	-0.041	-0.037	-0.010
NAICSA5	-0.024	-0.007	-0.017	0.000	-0.032
NAICSA6	-0.016	-0.047	-0.009	0.025	0.014
NAICSA7	0.042	-0.004	0.034	0.030	0.006
NAICSA8	0.050	0.011	0.019	0.000	0.030
NAICSA9	0.024	0.016	0.026	-0.005	0.013
NAICSA10	-0.007	0.051	0.018	0.045	0.026
NAICSA11	-0.012	0.018	-0.041	0.006	0.003
NAICSA12	0.001	-0.024	-0.018	-0.021	-0.056
NWRKLOC	-0.020	0.031	-0.021	-0.042	-0.006
EMPWLOC	0.010	0.026	0.033	0.042	0.053
EMPWTOT	-0.060	-0.001	-0.039	0.001	-0.013
JTYEAR	-0.002	0.013	0.016	0.040	0.042
NWHRS60	-0.036	0.041	-0.019	-0.001	0.016
SEASONAL	-0.009	0.030	0.033	0.053	0.044
PRECAREM	-0.093	-0.021	-0.057	-0.050	-0.040
UNION	0.015	0.013	0.012	0.017	0.003
QUARTER	0.053	0.068	0.086	0.030	0.047

	JOBSAT	ORGCOMIT	TURNOVER	LAYOFF	GENDERM
JOBSAT	1.000				
ORGCOMIT	0.677	1.000			
TURNOVER	-0.703	-0.681	1.000		
LAYOFF	-0.116	-0.111	0.081	1.000	
GENDERM	-0.065	-0.057	0.036	0.097	1.000
RACE2	-0.075	-0.108	0.100	0.048	0.002
AGE	0.209	0.350	-0.224	0.024	-0.058
EDUC	0.084	0.079	-0.072	0.060	-0.051
INCOME10	0.061	0.096	-0.080	0.067	0.058
MA	-0.022	-0.008	0.001	-0.024	0.006
ENC	0.001	0.017	-0.028	-0.017	-0.013
WNC	0.015	0.003	-0.035	-0.020	-0.025
SA	-0.002	0.033	0.028	-0.025	-0.003
ESC	-0.006	0.000	0.020	-0.025	0.019
WSC	0.018	-0.025	0.013	-0.028	-0.016
MNT	-0.001	-0.014	0.009	-0.002	0.013
PAC	-0.004	-0.018	0.008	0.113	0.017
SOC2NN2	0.105	0.073	-0.096	0.047	-0.128
SOC2NN3	-0.048	-0.108	0.093	-0.169	-0.028
SOC2NN4	-0.050	-0.101	0.061	-0.010	-0.028
SOC2NN5	-0.028	-0.018	0.042	-0.001	-0.187
SOC2NN7	0.034	0.027	-0.016	0.057	0.155
SOC2NN8	-0.050	0.023	-0.030	-0.031	0.159
SOC2NN9	0.036	0.057	-0.062	-0.002	0.081
SOC2NN10	-0.103	-0.091	0.060	0.032	0.178
NAICSA2	0.012	0.057	-0.016	0.104	0.145
NAICSA3	-0.129	-0.164	0.115	-0.001	0.015

	JOBSAT	ORGCOMIT	TURNOVER	LAYOFF	GENDERM
NAICSA4	0.008	0.010	-0.015	0.111	0.024
NAICSA5	-0.003	0.013	-0.023	0.037	-0.006
NAICSA6	-0.026	-0.023	0.009	0.043	0.060
NAICSA7	0.114	0.150	-0.127	0.009	-0.179
NAICSA8	0.025	0.008	-0.012	-0.091	-0.256
NAICSA9	-0.023	-0.142	0.103	-0.079	0.002
NAICSA10	-0.050	-0.025	0.059	-0.027	0.050
NAICSA11	0.062	0.096	-0.079	-0.120	0.093
NAICSA12	-0.012	-0.003	-0.007	0.012	0.078
NWRKLOC	-0.013	-0.010	-0.009	0.116	0.072
EMPWLOC	0.018	0.045	-0.082	0.170	0.013
EMPWTOT	-0.013	-0.012	-0.023	0.179	0.038
JTYEAR	0.133	0.236	-0.165	0.028	0.004
NWHRS60	0.039	0.140	-0.094	0.138	0.178
SEASONAL	-0.017	-0.017	0.005	-0.048	0.038
PRECAREM	-0.074	-0.095	0.053	0.040	0.041
UNION	0.058	0.108	-0.163	0.071	0.022
QUARTER	0.060	0.019	-0.016	-0.099	0.043

	RACE2	AGE	EDUC	INCOME10	MA
RACE2	1.000				
AGE	-0.125	1.000			
EDUC	-0.139	0.225	1.000		
INCOME10	-0.059	0.171	0.248	1.000	
MA	-0.056	0.030	0.030	0.018	1.000
ENC	-0.128	-0.008	-0.038	-0.045	-0.171
WNC	-0.102	0.035	-0.027	-0.026	-0.114
SA	0.092	0.024	0.013	-0.024	-0.207
ESC	0.009	-0.048	-0.014	-0.041	-0.107
WSC	0.021	-0.039	-0.017	-0.014	-0.124
MNT	-0.031	0.038	0.042	0.018	-0.104
PAC	0.165	-0.054	-0.021	0.056	-0.180
SOC2NN2	-0.068	0.153	0.523	0.101	-0.043
SOC2NN3	0.037	-0.189	-0.226	-0.131	-0.005
SOC2NN4	-0.018	-0.143	-0.174	-0.075	0.022
SOC2NN5	0.018	0.019	-0.127	-0.068	0.008
SOC2NN7	0.031	-0.024	-0.126	-0.040	-0.031
SOC2NN8	-0.010	-0.007	-0.136	-0.028	0.029
SOC2NN9	-0.017	-0.034	-0.153	-0.055	-0.042
SOC2NN10	0.048	-0.060	-0.247	-0.061	0.024
NAICSA2	-0.005	0.048	-0.099	0.020	-0.003
NAICSA3	-0.017	-0.114	-0.210	-0.078	-0.002
NAICSA4	0.006	0.030	0.024	0.019	0.003
NAICSA5	-0.041	0.028	0.055	0.111	0.051
NAICSA6	-0.050	0.005	0.168	0.069	-0.017
NAICSA7	-0.034	0.153	0.280	0.019	-0.036
NAICSA8	0.015	0.013	0.083	-0.014	0.032

	RACE2	AGE	EDUC	INCOME10	MA
NAICSA9	0.068	-0.259	-0.216	-0.116	0.005
NAICSA10	0.032	-0.005	-0.033	-0.048	-0.038
NAICSA11	-0.018	0.096	0.121	0.078	0.024
NAICSA12	0.060	0.041	-0.114	-0.011	0.021
NWRKLOC	0.006	0.096	0.047	0.035	-0.031
EMPWLOC	0.010	0.126	0.203	0.117	0.010
EMPWTOT	-0.025	0.095	0.174	0.089	-0.003
JTYEAR	-0.077	0.452	0.080	0.083	0.035
NWHRS60	-0.073	0.212	0.229	0.207	-0.030
SEASONAL	0.070	-0.093	-0.081	-0.075	-0.040
PRECAREM	0.071	-0.088	-0.088	-0.066	0.018
UNION	0.013	0.135	0.084	0.030	0.069
QUARTER	0.060	-0.006	-0.021	-0.020	-0.021

	ENC	WNC	SA	ESC	WSC
ENC	1.000				
WNC	-0.119	1.000			
SA	-0.217	-0.144	1.000		
ESC	-0.112	-0.074	-0.135	1.000	
WSC	-0.130	-0.086	-0.157	-0.081	1.000
MNT	-0.109	-0.073	-0.132	-0.068	-0.079
PAC	-0.188	-0.125	-0.228	-0.118	-0.136
SOC2NN2	-0.009	-0.017	0.009	-0.029	0.034
SOC2NN3	0.063	0.001	-0.022	-0.013	-0.007
SOC2NN4	-0.002	-0.004	-0.020	0.005	0.014
SOC2NN5	-0.007	0.016	0.004	0.037	-0.003
SOC2NN7	-0.001	0.019	-0.033	-0.015	-0.054
SOC2NN8	-0.009	-0.033	0.023	0.029	-0.042
SOC2NN9	0.070	0.064	0.007	-0.024	-0.032
SOC2NN10	-0.029	0.032	0.026	0.000	0.018
NAICSA2	0.079	0.076	-0.033	-0.026	-0.054
NAICSA3	0.020	-0.013	-0.052	0.015	0.109
NAICSA4	-0.016	-0.001	0.003	-0.028	-0.018
NAICSA5	-0.044	-0.004	0.008	0.002	0.008
NAICSA6	-0.023	-0.006	0.002	0.029	-0.002
NAICSA7	0.021	0.001	-0.022	-0.046	0.072
NAICSA8	0.028	-0.010	-0.033	0.027	-0.032
NAICSA9	-0.028	-0.025	0.006	-0.028	-0.027
NAICSA10	-0.025	-0.008	0.095	0.084	-0.031
NAICSA11	-0.051	-0.026	0.060	-0.006	0.004
NAICSA12	-0.007	0.014	0.063	-0.005	-0.051
NWRKLOC	-0.011	0.005	0.037	0.017	-0.034

	ENC	WNC	SA	ESC	WSC
EMPWLOC	0.031	0.013	-0.012	-0.027	-0.038
EMPWTOT	-0.006	0.009	0.031	-0.015	-0.033
JTYEAR	0.032	0.069	-0.064	-0.044	-0.049
NWHRS60	-0.025	-0.024	0.064	0.010	0.057
SEASONAL	-0.017	0.067	-0.048	0.038	-0.006
PRECAREM	0.014	-0.036	-0.037	-0.010	0.001
UNION	0.075	-0.021	-0.114	-0.081	-0.091
QUARTER	-0.031	0.027	0.019	0.071	-0.057

	MNT	PAC	SOC2NN2	SOC2NN3	SOC2NN4
MNT	1.000				
PAC	-0.115	1.000			
SOC2NN2	0.069	-0.003	1.000		
SOC2NN3	-0.056	0.033	-0.289	1.000	
SOC2NN4	-0.009	0.008	-0.202	-0.123	1.000
SOC2NN5	-0.043	-0.020	-0.282	-0.172	-0.120
SOC2NN7	0.064	0.046	-0.122	-0.074	-0.052
SOC2NN8	0.008	0.004	-0.135	-0.082	-0.057
SOC2NN9	-0.002	-0.033	-0.135	-0.082	-0.057
SOC2NN10	-0.017	-0.035	-0.187	-0.113	-0.079
NAICSA2	0.014	-0.041	-0.074	-0.134	-0.068
NAICSA3	-0.006	-0.027	-0.203	-0.094	0.421
NAICSA4	-0.002	0.039	0.009	-0.073	0.006
NAICSA5	-0.016	-0.024	-0.104	-0.114	0.067
NAICSA6	0.044	-0.022	0.211	-0.110	-0.071
NAICSA7	0.011	0.007	0.383	-0.085	-0.117
NAICSA8	-0.008	-0.014	0.171	0.094	-0.119
NAICSA9	-0.032	0.085	-0.199	0.474	-0.011
NAICSA10	-0.053	-0.011	-0.085	0.086	0.047
NAICSA11	0.000	-0.007	-0.008	0.025	-0.071
NAICSA12	-0.021	-0.031	-0.139	-0.082	-0.060
NWRKLOC	0.037	-0.034	-0.033	-0.095	0.062
EMPWLOC	0.014	-0.006	0.154	-0.155	-0.108
EMPWTOT	0.032	-0.026	0.063	-0.140	0.014
JTYEAR	0.013	0.002	0.115	-0.112	-0.093
NWHRS60	0.015	-0.074	0.105	-0.218	-0.133
SEASONAL	-0.044	0.065	0.006	0.052	0.011

	MNT	PAC	SOC2NN2	SOC2NN3	SOC2NN4
PRECAREM	-0.028	0.076	-0.026	-0.014	0.025
UNION	-0.019	0.126	0.115	-0.033	-0.104
QUARTER	0.018	0.001	0.001	0.036	0.037

	SOC2NN5	SOC2NN7	SOC2NN8	SOC2NN9	SOC2NN10
SOC2NN5	1.000				
SOC2NN7	-0.072	1.000			
SOC2NN8	-0.080	-0.035	1.000		
SOC2NN9	-0.080	-0.035	-0.038	1.000	
SOC2NN10	-0.111	-0.048	-0.053	-0.053	1.000
NAICSA2	-0.005	-0.021	0.094	0.384	0.015
NAICSA3	0.030	-0.066	-0.056	-0.003	0.147
NAICSA4	0.058	-0.031	0.149	-0.034	-0.043
NAICSA5	0.160	-0.016	-0.028	-0.054	-0.074
NAICSA6	-0.027	-0.047	-0.051	-0.041	-0.071
NAICSA7	-0.078	-0.068	-0.047	-0.080	-0.107
NAICSA8	-0.019	-0.074	-0.082	-0.070	-0.101
NAICSA9	-0.058	-0.058	-0.048	-0.050	-0.028
NAICSA10	-0.009	-0.021	0.067	-0.019	-0.030
NAICSA11	0.001	-0.029	0.065	0.001	-0.056
NAICSA12	0.038	-0.028	-0.036	-0.040	0.528
NWRKLOC	0.007	-0.007	0.018	-0.008	-0.009
EMPWLOC	-0.017	-0.091	-0.041	0.041	0.004
EMPWTOT	0.013	-0.139	-0.006	0.011	-0.020
JTYEAR	-0.013	-0.007	0.025	-0.007	0.010
NWHRS60	-0.106	-0.002	0.031	0.049	0.023
SEASONAL	-0.026	0.172	-0.051	-0.051	0.020
PRECAREM	-0.021	0.158	0.005	0.001	0.071
UNION	-0.067	0.033	0.055	0.025	0.060
QUARTER	-0.017	-0.027	0.031	0.059	-0.054

	NAICSA2	NAICSA3	NAICSA4	NAICSA5	NAICSA6
NAICSA2	1.000				
NAICSA3	-0.126	1.000			
NAICSA4	-0.058	-0.065	1.000		
NAICSA5	-0.092	-0.102	-0.047	1.000	
NAICSA6	-0.088	-0.099	-0.046	-0.072	1.000
NAICSA7	-0.136	-0.152	-0.070	-0.111	-0.107
NAICSA8	-0.140	-0.157	-0.072	-0.114	-0.110
NAICSA9	-0.109	-0.122	-0.056	-0.089	-0.086
NAICSA10	-0.087	-0.098	-0.045	-0.071	-0.069
NAICSA11	-0.098	-0.110	-0.051	-0.080	-0.077
NAICSA12	-0.069	-0.077	-0.035	-0.056	-0.054
NWRKLOC	0.024	0.073	0.079	0.093	0.009
EMPWLOC	0.193	-0.086	0.070	0.006	-0.040
EMPWTOT	0.096	0.044	0.086	0.102	-0.028
JTYEAR	0.042	-0.074	0.026	-0.015	0.014
NWHRS60	0.106	-0.073	0.070	0.078	0.059
SEASONAL	-0.060	-0.053	-0.018	-0.071	-0.064
PRECAREM	-0.041	0.006	0.005	-0.009	-0.053
UNION	-0.074	-0.085	-0.041	-0.097	-0.098
QUARTER	0.045	-0.037	-0.040	0.018	-0.026

	NAICSA7	NAICSA8	NAICSA9	NAICSA10	NAICSA11
NAICSA7	1.000				
NAICSA8	-0.170	1.000			
NAICSA9	-0.132	-0.136	1.000		
NAICSA10	-0.106	-0.109	-0.085	1.000	
NAICSA11	-0.119	-0.123	-0.095	-0.076	1.000
NAICSA12	-0.083	-0.085	-0.067	-0.053	-0.060
NWRKLOC	-0.092	-0.034	-0.103	-0.073	0.075
EMPWLOC	0.039	0.076	-0.138	-0.144	0.024
EMPWTOT	-0.021	-0.010	-0.132	-0.148	0.073
JTYEAR	0.115	-0.025	-0.114	-0.042	0.034
NWHRS60	0.082	-0.106	-0.219	-0.044	0.080
SEASONAL	0.112	-0.059	0.065	0.111	-0.061
PRECAREM	0.002	-0.055	-0.040	0.091	-0.027
UNION	0.240	-0.025	-0.100	-0.091	0.174
QUARTER	-0.044	0.003	0.061	0.017	0.032

	NAICSA12	NWRKLOC	EMPWLOC	EMPWTOT	JTYEAR
NAICSA12	1.000				
NWRKLOC	0.052	1.000			
EMPWLOC	0.070	0.195	1.000		
EMPWTOT	0.076	0.589	0.627	1.000	
JTYEAR	0.091	0.011	0.084	0.064	1.000
NWHRS60	0.024	0.124	0.174	0.196	0.121
SEASONAL	0.004	-0.078	-0.066	-0.136	-0.063
PRECAREM	0.055	0.030	-0.105	-0.067	-0.088
UNION	0.168	0.051	0.135	0.135	0.247
QUARTER	-0.011	-0.044	-0.046	-0.041	0.004

	NWHRS60	SEASONAL	PRECAREM	UNION	QUARTER
	_________	_________	_________	_________	_________
NWHRS60	1.000				
SEASONAL	-0.147	1.000			
PRECAREM	-0.147	0.243	1.000		
UNION	0.096	0.022	-0.019	1.000	
QUARTER	-0.062	0.024	0.012	-0.049	1.000
