# Supplementary material for: Organizational Downsizing, Work Conditions, and Employee Outcomes: Identifying Targets for Workplace Intervention among Survivors
Source: Int J Environ Res Public Health. 2020 Jan 22;17(3):719. doi: 10.3390/ijerph17030719 (PMC7037986; doi:10.3390/ijerph17030719)
Supplement: Supplementary file 1 [file ijerph-17-00719-s001.zip › Appendix S2.pdf]

Appendix S2

This appendix contains the standardized factor loadings from the confirmatory factor analysis of the 27 multi-item constructs shown in Figure 1 of the article. The downsizing and inability to unwind variables were not included because they were assessed with single items. See the Analysis section in the article for more detail on this analysis. All standardized factor loadings reported below are significant at $p < .001$.

Standardized Factor Loadings

Constructs / Items	Standardized Factor Loadings
**Work Demands**	
Item 1	.84
Item 2	.88
Item 3	.89
Item 4	.74
Item 5	.84
Item 6	.66
**Role Conflict**	
Item 1	.87
Item 2	.86
Item 3	.90
**Role Ambiguity**	
Item 1	.78
Item 2	.84
Item 3	.84
Item 4	.88
**Work Autonomy**	
Item 1	.70
Item 2	.76
Item 3	.86
Item 4	.83
Item 5	.86
Item 6	.85
**Supervisor Aggression**	
Item 1	.87
Item 2	.94
Item 3	.85
**Coworker Aggression**	
Item 1	.91
Item 2	.91
Item 3	.77

**Friendship Formation**	
Item 1	.87
Item 2	.95
Item 3	.93
**Dysfunctional Leadership**	
Item 1	.75
Item 2	.86
Item 3	.89
Item 4	.79
Item 5	.83
Item 6	.86
Item 7	.80
Item 8	.85
Item 9	.89
Item 10	.79
**Distributive Justice**	
Item 1	.90
Item 2	.94
Item 3	.94
Item 4	.92
**Promotion Opportunity**	
Item 1	.76
Item 2	.82
Item 3	.87
**Job Insecurity**	
Item 1	.83
Item 2	.79
Item 3	.89
**Employment Insecurity**	
Item 1	.89
Item 2	.78
Item 3	.90
**Negative Work Rumination**	
Item 1	.81
Item 2	.91
Item 3	.91
Item 4	.92

**Physical Work Fatigue**	
Item 1	.86
Item 2	.86
Item 3	.93
Item 4	.90
Item 5	.93
Item 6	.89
**Mental Work Fatigue**	
Item 1	.92
Item 2	.90
Item 3	.95
Item 4	.89
Item 5	.95
Item 6	.89
**Emotional Work Fatigue**	
Item 1	.89
Item 2	.96
Item 3	.95
Item 4	.86
Item 5	.93
Item 6	.92
**Depression**	
Item 1	.85
Item 2	.67
Item 3	.70
**Anxiety**	
Item 1	.75
Item 2	.66
Item 3	.82
**Anger**	
Item 1	.72
Item 2	.61
Item 3	.82
**Happiness**	
Item 1	.76
Item 2	.93
Item 3	.86

**Confidence**	
Item 1	.80
Item 2	.62
Item 3	.77
**Vigor**	
Item 1	.79
Item 2	.80
Item 3	.86
**Physical Health**	
Item 1	.84
Item 2	.79
**Mental Health**	
Item 1	.95
Item 2	.78
**Job satisfaction**	
Item 1	.93
Item 2	.95
Item 3	.93
**Organizational Commitment**	
Item 1	
Item 2	.88
Item 3	.96
	.88
**Turnover Intentions**	
Item 1	.93
Item 2	.94
Item 3	.87
